# Tryptophan Metabolism: A Versatile Area Providing Multiple Targets for Pharmacological Intervention

**DOI:** 10.32527/2019/101415

**Published:** 2019

**Authors:** Abdulla Abu-Bakr Badawy

**Affiliations:** Formerly School of Health Sciences, Cardiff Metropolitan University, Cardiff, Wales, UK

**Keywords:** immunotherapy, indoleamine 2,3-dioxygenase, inflammation, kynurenine pathway, major depressive disorder, neurological disease, plasma free tryptophan, serotonin pathway, tryptophan 2,3-dioxygenase, tumoral immune escape

## Abstract

The essential amino acid *L*-tryptophan (Trp) undergoes extensive metabolism along several pathways, resulting in production of many biologically active metabolites which exert profound effects on physiological processes. The disturbance in Trp metabolism and disposition in many disease states provides a basis for exploring multiple targets for pharmaco-therapeutic interventions. In particular, the kynurenine pathway of Trp degradation is currently at the forefront of immunological research and immunotherapy. In this review, I shall consider mammalian Trp metabolism in health and disease and outline the intervention targets. It is hoped that this account will provide a stimulus for pharmacologists and others to conduct further studies in this rich area of biomedical research and therapeutics.

## 1 Introduction

The essential amino acid *L*-tryptophan (Trp) is unique among amino acids in the wide range of biologically active metabolites produced along its 4 degradative pathways. Trp metabolites influence body physiology at multiple levels and it is therefore no surprise that Trp research extends across many scientific disciplines and medical specialties (Table 1). These include: (1) Basic sciences (mammalian, insect and plant biochemistry, behavioural science, immunology, neurochemistry, nutrition science, pharmacology, physiology); (2) Medical specialties (cardiology, diabetes, gastroenterology, hepatology, obstetrics & gynaecology, oncology, ophthalmology, parasitology, rheumatology, urology, virology, veterinary medicine); (3) Psychiatry (alcoholism, anxiety, depression, drug dependence, obsessive-compulsive disorder, schizophrenia); (4) Neurological disease (Alzheimer’s disease, chronic brain injury, Huntington’s disease, stroke). For this reason, an International Society for Tryptophan Research (ISTRY) dedicated to studying this amino acid exists and holds triennial conferences (https://www.istry.org/).

Trp research has been actively practiced in Egypt from the 1960s on, mainly in relation to *Schistosoma* infestation, by the group of Dr Gamal Abdel-Tawab at the National Research Centre in Gizeh and the Medical Research Institute in Alexandria (see, e.g., [1–4]). Trp research is a fruitful area that never disappoints. The eminent Japanese scientist, Professor Osamu Hayaishi, discoverer of indoleamine 2,3-dioxygenase, testifies to this fact in the title of his paper “My life with tryptophan: never a dull moment” [5]. I have been studying Trp over the past 48 years and have never once been disappointed by the results. I hope that the readership of this journal will find the following account a stimulus for exploring and enriching our knowledge of this amino acid in health and disease. In the following text, a description of plasma Trp disposition will be followed by an account of each of the Trp-degradative pathways. Each account will include the general biochemical features and control of the pathway and, where appropriate, a discussion of targets for pharmacological intervention in relation to disease states.

Of the 4 known pathways of Trp metabolism in mammals, 3 are of minor quantitative significance, namely the hydroxylation (serotonin and melatonin), decarboxylation (tryptamine), and transamination (indolepyruvic acid) pathways, with each contributing ~ 1% to overall dietary Trp degradation. Functionally, these 3 pathways are no less important than the 4th, the oxidative (kynurenine) pathway (KP), which accounts for ~ 95% of dietary Trp degradation [6]. Although Trp is essential for protein synthesis, dietary Trp contributes nothing to this process in the nitrogen balance state, wherein Trp released by protein degradation is reutilised for protein synthesis [7]. Consequently, dietary Trp is totally available for metabolism [7].

## 2 Plasma Tryptophan Disposition

### 2.1 General features and control

After absorption following dietary protein digestion, Trp exists in plasma largely (90–95%) bound to albumin, with the remaining 5–10% being free and hence immediately available for tissue uptake and metabolism. Equilibration between free and albumin-bound Trp is, however, rapid, such that a sustained increase in free [Trp] coupled with continued tissue uptake results in depletion of total (free + albumin-bound) [Trp]. This is best seen in rats treated with a single acute dose of sodium salicylate or ethanol [8]. The physiological binder of Trp is albumin, whereas the physiological displacers of albumin-bound Trp are non-esterified fatty acids (NEFA). Trp binding is usually expressed as the % free Trp (100 × [free Trp]/[total Trp]. Free Trp is best isolated from plasma or serum by ultrafiltration, rather than by equilibrium dialysis, as the latter is less accurate. Ultrafiltration should be performed using freshly isolated plasma or serum and never after frozen storage and subsequent thawing, as frozen storage increases Trp binding to albumin, thereby giving an artifactually low free [Trp] [9]. Because of the above rapid equilibration, accurate interpretation of changes in plasma Trp disposition necessitates determination of both free and total [Trp]. Equally important is that these simultaneous determinations can help establish the baseline Trp disposition status and its biological determinants [8]. Free and total Trp are now determined by HPLC (high-performance liquid chromatography) or GC (gas chromatography), with or without mass spectrometry. However, in the absence of these techniques, Trp can be measured fluorimetrically by the method of Denckla and Dewey [10], as modified by Bloxam and Warren [11] to avoid errors in the former.

Table 2 lists the number of conditions influencing plasma Trp disposition and the mechanisms involved [12]. Important points to note from this Table are the following. Induction of liver Trp 2,3-dioxygenase (TDO) or extrahepatic indoleamine 2,3-dioxygenase (IDO) should decrease both free and total [Trp] by similar proportions without altering the % free Trp. Similarly, TDO inhibition should increase both free and total [Trp] without altering the % free Trp. Other changes in free [Trp] and the % free Trp occur in parallel when Trp binding is altered. Binding is increased when NEFA are decreased by insulin, nicotinic acid or other antilipolytic agents, but is decreased by displacement from albumin-binding sites by NEFA and agents acting via NEFA, by direct displacement, e.g. by salicylate, or if [albumin] is decreased, e.g. in pregnancy and liver and kidney diseases.

Plasma free Trp can also be influenced by diet. Dietary proteins and lipids will increase it, whereas dietary carbohydrates will decrease it [8], with proteins providing Trp, lipids providing NEFA and inhibiting liver TDO, and carbohydrates acting via insulin. Thus, assessing the plasma free Trp status should take into consideration the potential effects of food and drugs. To avoid the influence of recent food intake, free and total [Trp] should be determined in plasma or serum of overnight fasting subjects.

### 2.2 Pharmacological targeting

As far as I could ascertain, pharmacological intervention to alter plasma Trp availability has been limited to the use of Trp mainly in the treatment of depression in relation to the serotonin deficiency in this condition. This will be discussed further under **3** (the hydroxylation or serotonin pathway). Targeting Trp availability to tumors has recently been proposed [13] to overcome tumoral immune escape. Here, decreasing plasma free [Trp] using antilipolytic agents, albumin infusions or both has been suggested. This will be discussed further under **6** (the oxidative or kynurenine pathway).

## 3 The Hydroxylation or Serotonin Pathway

### 3.1 General features and control

**Figure 1:**
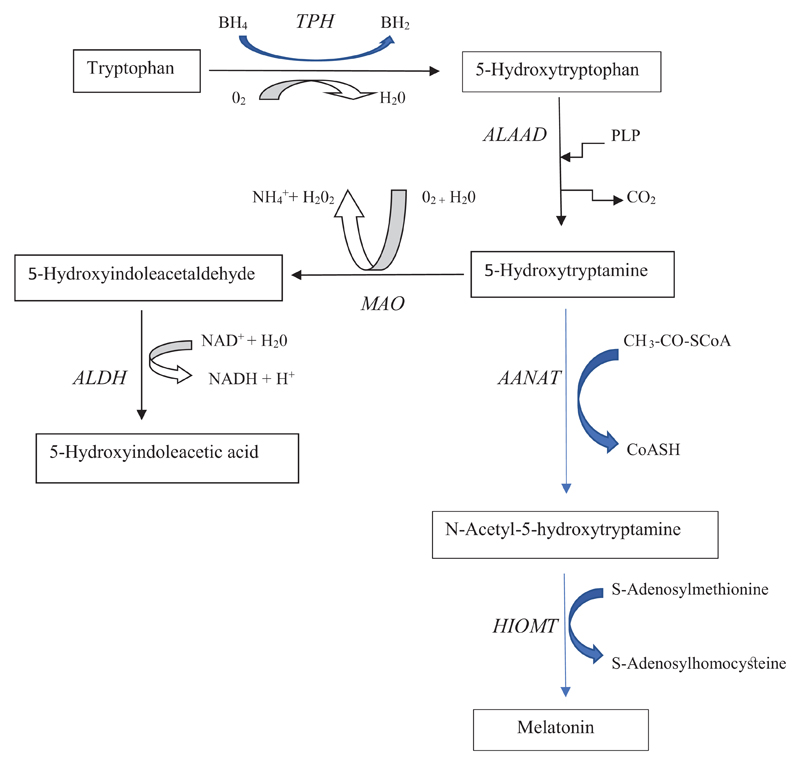
The hydroxylation or serotonin pathway in brain and melatonin pathway in pineal. Abbreviations used are: AANAT (arylalkylamine N-acetyltransferase), ALAAD (aromatic *L*-amino acid decarboxylase), ALDH (aldehyde dehydrogenase), BH_2_ and BH_4_ (dihydro- and tetrahydro-biopterin), HIOMT (hydoxyindole-*O*-methyltransferase), MAO (monoamine oxidase), PLP (pyridoxal 5’-phosphate), TPH (tryptophan hydroxylase).

Serotonin synthesis is a 2-step process (Figure 1). First, Trp is hydroxylated to 5-hydroxytryptophan (5-HTP) by Trp hydroxylase (TPH). This is then followed by decarboxylation of 5-HTP to 5-hydroxytryptamine (5-HT) or serotonin. The serotonin pathway exists in brain, gastrointestinal tract and pineal gland. 5-HT is further converted to melatonin in the pineal gland and the periphery. TPH exists in 2 isoforms: TPH1 in the periphery and TPH2 in the central nervous system (CNS). 5-HT is metabolised mainly by oxidation to 5-hydroxindoleacetaldehyde by monoamine oxidase (MAO) and this is followed by further oxidation to 5-hydroxyindoleacetic acid (5-HIAA), the main urinary serotonin metabolite, by the action of aldehyde dehydrogenase (ALDH). Not shown in Figure 1 is the reduction of 5-hydroxindoleacetaldehyde to 5-hydroxytryptophol by the action of aldehyde reductase. This is a minor pathway of serotonin metabolism, which can be enhanced by alcohol consumption and metabolism providing adequate amounts of the NAD(P)H cofactor for the reductase. 5-Hydroxytryptophol is thought to promote sleep. In the pineal gland and elsewhere, 5-HT is converted to melatonin in a 2-step process: N-acetylation to N-acetyl serotonin followed by *o* methylation of the 5 hydroxy group of the latter to melatonin.

In rat and other experimental animal brains, TPH is the rate-limiting enzyme in cerebral 5-HT synthesis. However, whereas TPH activity can be influenced by factors such as phosphorylation of the enzyme and level of the cerebral cofactor tetrahydrobiopterin (BH_4_), brain [Trp] is the major determinant of TPH activity, because the enzyme exists unsaturated (≤ 50%) with its Trp substrate [14], i.e. the physiological [Trp] is lower than the K_*m*_ of TPH. Thus, minor fluctuations in [Trp] in brain or its availability in the circulation can have a significant impact on cerebral 5-HT synthesis.

Excess Trp can, however, decrease serotonin synthesis by substrate inhibition of TPH. Such inhibition has been demonstrated *in vitro* when TPH activity was determined by measuring brain [5-HTP] after ALAAD inhibition but in the presence of the natural BH_4_ cofactor and not its analogue dimethyl tetrahydrobiopterin [15], and *in vivo* after administration of large doses of Trp [16]. Substrate (Tyr) inhibition of brain tyrosine hydroxylase activity has also been similarly demonstrated both *in vitro* [17] and *in vivo* after administration of various doses of Tyr [18]. Substrate inhibition of TPH *in vivo* is also suggested by the finding that, whereas rat brain [Trp] continues to rise with increasing dosage, the increases in brain [5-HT] and [5-HIAA] cease to rise and begin to decrease with doses of Trp of 50 mg/kg and above [19]. In general, the increase in brain [5-HT] after Trp loading rarely reaches, and certainly does not exceed, twofold.

Whereas assessing cerebral 5-HT synthesis in rats can be achieved by direct measurement of brain levels of Trp, 5-HT and the major serotonin metabolite 5-HIAA and by estimating the rate of hydroxylation of Trp to 5-HTP *in vivo* by measuring brain levels of the latter following inhibition of aromatic *L*-amino acid decarboxylase (ALAAD) activity in brain, e.g. by compound NSD-1015, these techniques cannot be used in humans for obvious reasons. As brain [Trp] is the major determinant of TPH activity and hence brain 5-HT synthesis, assessing the likely changes in brain [Trp] in humans is currently performed by measuring the ratio of plasma [Trp] to the sum of 5 competing amino acids [CAA] which share with Trp the same cerebral uptake mechanism. The change in this ratio is considered a reasonable reflection of the likely changes in brain [Trp] and hence 5-HT synthesis. The 5 competing amino acids are the 3 branched-chain amino acids (BCAA) Val, Leu and Ile, and the 2 aromatic amino acids Phe and Tyr. Both the free and total [Trp]/[CAA] ratios should be determined. Utilising this principle, brain 5-HT can be acutely decreased or increased by the techniques of acute Trp depletion (ATD) or loading (ATL) respectively [20, 21], which are widely used to manipulate serotonin levels in diagnostic and research studies in psychiatric and other conditions associated with serotonin dysfunction. In the original method of Young *et al* [20], the high content of BCAA led to decreased specificity towards serotonin. This has been remedied by lowering the BCAA content by 40% [21]. Although the second enzyme of 5-HT synthesis, ALAAD, is thought not to be rate-limiting in rat brain, it may be limiting in human brain, where its activity is low [22]. ALAAD is a pyridoxal 5’-phosphate (PLP)-dependent enzyme and it is therefore possible that subjects with vitamin B_6_ deficiency could be at risk of becoming serotonin-deficient. B_6_ deficiency could be nutritional or drug-induced, e.g. by hydrazine compounds (e.g. benserazide and carbidopa) and oestrogens.

Melatonin is synthesized in the pineal gland, retina and various other brain structures, the gastrointestinal tract and many other peripheral tissues [23] and exerts a wide range of effects, including regulation of the sleep-wakefulness cycle, modulation of immune function, the endocrine system and reproduction, free radical scavenging (antioxidant effect) and regulation of the mental state and behaviour [24]. Pineal gland calcification results in decreased pineal melatonin production and is associated with neurological diseases and other pathologies [25]. Melatonin is synthesized from Trp via serotonin. Pineal synthesis occurs at night. Of the 2 steps of synthesis from serotonin, the first, catalysed by AANAT (arylalkylamine N-acetyl transferase) is rate-limiting. In the chicken pineal and retina, the mRNAs encoding AANAT, HIOMT and TPH are expressed in a day/night rhythm, with the rhythm in the pineal persisting under conditions of constant darkness [26]. Trp loading increases melatonin synthesis in the rat pineal by up to 100% [27]. As this is also the maximum elevation of brain serotonin levels after Trp loading (because of substrate inhibition of TPH by excess Trp, see above) and as serotonin is the precursor of melatonin, it may be concluded that synthesis of the latter from Trp is subject to the same TPH control mechanisms applicable to serotonin synthesis.

### 3.2 Pharmacological targeting

The serotonin pathway has been a target for pharmacotherapy of conditions associated with serotonin deficiency or dysfunction (Table 3). The monoamine hypothesis of affective disorders that postulates a deficiency in one or more monoamines (serotonin, dopamine and/or noradrenaline) formed the basis of the first pharmacotherapy of these conditions, with major depressive disorder (MDD) having received the greatest attention. To ensure adequate levels of cerebral monoamines, preventing their degradation was the first adopted approach in pharmacotherapy. This was achieved by using and developing monoamine oxidase inhibitors (MAOI) and inhibitors of monoamine reuptake at the synaptic cleft, the tricyclic antidepressants. There is a wide array of antidepressant drugs of different chemical structures and pharmacological profiles in current use. Those related to serotonin function include selective serotonin-reuptake inhibitors (SSRIs), serotonin-noradrenaline reuptake inhibitors, serotonin antagonists, MAOIs and some tricyclic antidepressants.

MAO-A is the isoform of MAO that preferentially deaminates serotonin, adrenaline, noradrenaline and melatonin. Different trace amines are deaminated by MAO-A and MAO-B. Dopamine is equally deaminated by both isoforms. In addition to their use in depression, MAOIs are also used in Parkinson’s disease, panic disorder and post-traumatic stress disorder. A recent development in MAO targeting is in prostate cancer, where the A form is highly expressed [28, 29]. Targeting MAOs with multipotent ligands has been suggested as a potential strategy in the search for new drugs to treat neurodegenerative diseases [30].

The B form of MAO has been implicated in the neurotoxicity and Parkinson’s disease-like symptoms in subjects receiving MPTP (1-methyl-4-phenyl-1,2,3,6-tetrahydropyridine): a by-product in the synthesis of a mepyridine analogue first used as a substitute for heroin [31, 32]. MPTP itself is not neurotoxic, but is converted into toxic pyridinium metabolites by MAO-B.

Inhibition of MAO-B by deprenyl prevents the toxicity, and the abundance of MAO-B in dopaminergic neurons explains the high susceptibility of these neurons to MPTP toxicity (see [31] and references cited therein).

Targeting ALAAD is currently the mechanism of therapy of Parkinson’s disease, which involves dopamine deficiency. To ensure that adequate amounts of the dopamine precursor *L-*Dopa (3,4-dihydroxyphenylalanine) reach the brain, it is administered jointly with inhibitors of peripheral ALAAD (such as carbidopa and benserazide) to prevent its decarboxylation to dopamine in the periphery. Other ALAAD inhibitors include 3-hydroxybenzylhydrazine and *L*-α-methyldopa.

Targeting TPH2 has more recently been proposed to enhance brain serotonin synthesis in major depressive disorder [33]. The authors proposed approaches including transcriptional activation of the TPH2 gene, gene therapy, post-translational modifications (e.g. by phosphorylation), and use of 5-HT precursors. The first two approaches may be effective only in the long-term and are not without risk, whereas the third can only have a moderate effect (see [34] and references cited therein). The key to boosting central serotonin synthesis in the short term is in the TPH2 kinetic properties and especially Trp availability [34]. One of the early therapies of depression was oral Trp administration. Results of clinical trials with Trp have been equivocal and it appears that, at best, Trp may exert a moderate antidepressant effect in mildly depressed patients (for references, see [34]). The reason for the poor efficacy of Trp is almost certain to be its increased hepatic degradation by TDO leading to decreased availability to the brain. This decreased availability is suggested by the decrease in plasma [Trp] and in the [Trp]/[CAA] ratio in depressed patients (for references, see [34]). As will be discussed below, an inverse relationship exists between liver TDO and brain [Trp] and 5-HT synthesis. Liver TDO is likely to be induced in MDD, at least in ~50% of patients, in whom cortisol is elevated. TDO can also be activated in MDD by other mechanisms, e.g. raised peripheral catecholamines enhancing lipolysis, thereby increasing free Trp entry into liver and activating TDO by a substrate-type mechanism [34] (see below). For Trp to be an effective antidepressant, it has been suggested that it is administered together with a liver TDO inhibitor [35, 36]. Subsequent studies showed that a large number of antidepressant drugs of various chemical structures and pharmacological profiles also inhibit TDO (Trp pyrrolase) activity (see [34] and references cited therein). TDO inhibition will be further discussed in **6** (the oxidative or kynurenine pathway). In 8 out of 13 clinical trials, Trp was shown to potentiate the antidepressant effects of MAOIs and tricyclic antidepressants (for references, see [37]). Many important features of the role of Trp in MDD are discussed in [34], including comparative TDO-inhibitory potencies of antidepressants, the effects of mood stabilisers, augmenters and adjunctive therapies (Li^+^, carbamazepine, allopurinol, nicotinamide, oestrogens, progesterone, salicylate, the β-adrenoceptor blocker pindolol and T_3_) on Trp metabolism, the optimal Trp dosages, the delayed therapeutic response to antidepressants and the effects of the latter on circulating cortisol. The role of cytokines and inflammation in depression is also briefly discussed, though, in the author’s opinion, inflammation does not play a role in the impaired availability of circulating Trp to the brain for serotonin synthesis in MDD, even if it contributes significantly to the psychopathology of depression. This will be discussed further in 6 below.

The serotonin pathway in the periphery plays an important role in certain conditions, e.g., osteoporosis, irritable bowel syndrome, carcinoid syndrome, obesity, ulcerative colitis and pulmonary arterial hypertension. Current and potential inhibitors of TPH1 have been reviewed [38].

The important physiological roles of melatonin render it an attractive target for pharmacotherapy of certain conditions in addition to sleep disturbances and jet lag. Such conditions include autism spectrum disorder, where a defective melatonin synthesis due to impaired AANAT and HIOMT in pineal, gut and platelets involves post-translational and post-transcriptional mechanisms [39], cancer, where melatonin can influence many of its hallmarks [40] and protection of mitochondria [41]. AANAT is the enzyme involved in the diurnal rhythm of pineal melatonin synthesis and N-bromoacetyltryptamine has recently been shown to be a potent inhibitor of this enzyme and suggested for use to dissect the role of melatonin in the circadian rhythm and a potential lead compound for therapeutic use in mood and sleep disorders [42].

## 4 The Decarboxylation or Tryptamine Pathway

### 4.1 General features and control

**Figure 2:**
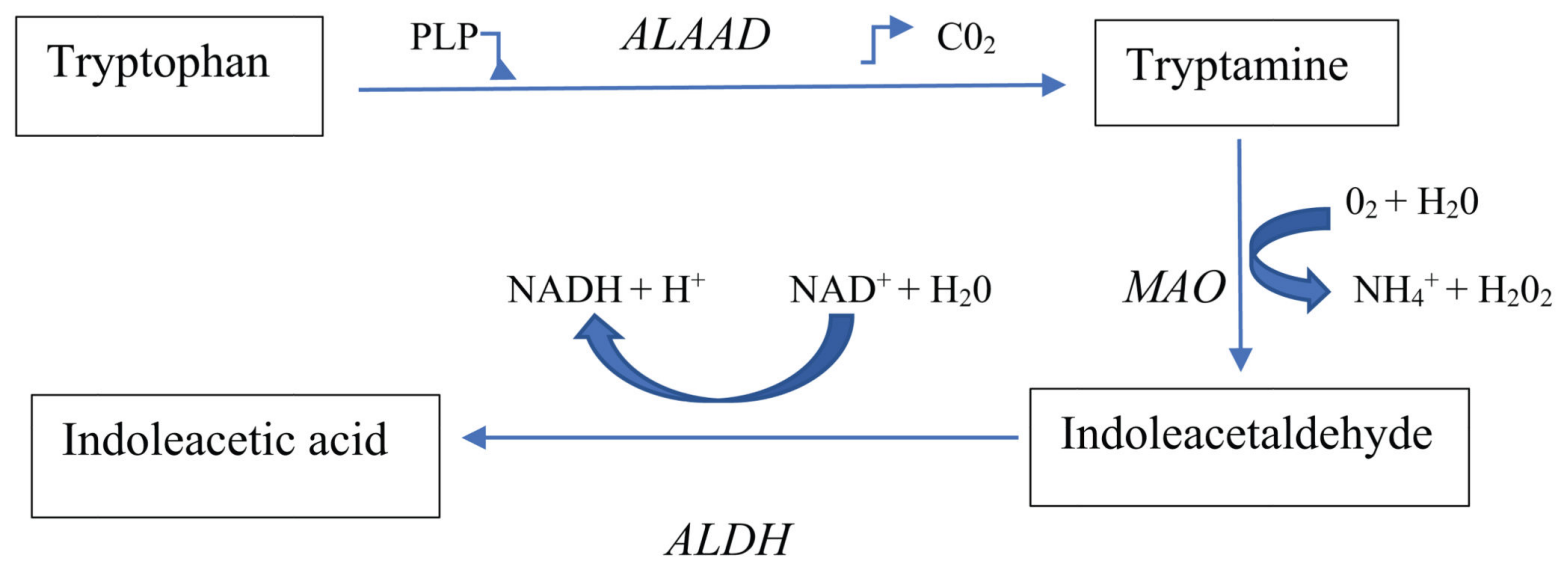
The decarboxylation or tryptamine pathway. Abbreviations used are: ALAAD (aromatic *L*-amino acid decarboxylase), ALDH (aldehyde dehydrogenase), MAO (monoamine oxidase), NAD(H) [oxidized and (reduced) nicotinamide-adenine dinucleotide], PLP (pyridoxal 5’-phosphate).

Aromatic *L*-amino acid decarboxylase (ALAAD) decarboxylates amino acids to their respective amines and intermediates of monoamine biosynthetic pathways to their respective monoamines, e.g. Trp to tryptamine, Tyr to tyramine, Phe to phenylethylamine, 5-HTP to 5-HT and *L*-Dopa to dopamine. The enzyme uses pyridoxal 5’-phosphate (PLP) as cofactor and releases CO_2_ (Figure 2). It is therefore controlled by availability of the cofactor and of the Trp substrate. Decarboxylation of Trp to tryptamine occurs in the periphery and the CNS, where tryptamine has a very rapid turnover rate and is thought to fulfil the criteria for a neurotransmitter and also a modulator of monoamine function [43], thereby implicating it in various psychiatric conditions, including hepatic encephalopathy [43, 44]. Patients with hepatic coma exhibit a greater tryptamine turnover, as assessed by levels of the metabolite indoleacetic acid (IAA) [45]. Urinary excretion of IAA is elevated in some conditions, including diabetes, neuromuscular disorders, amyotrophic lateral sclerosis and idiopathic sprue, but not in many others [46]. Brain [tryptamine] in humans and several animal species is generally, however, in the sub-micromolar range, but can be dramatically increased by MAO inhibition (see [43] and references cited therein). The decarboxylation pathway contributes little to overall Trp degradation [47].

The pathway also exists in the gastrointestinal tract. Thus, it has been known for some time that part of brain tryptamine is of intestinal bacterial origin [48]. More recently, Williams *et al* [49] demonstrated at least one of two bacteria encoding Trp decarboxylase in guts of 10% of the human population: the common gut firmicute *Clostridium sporogenes* and *Ruminococcus gnavus*.

### 4.2 Pharmacological targeting

Halogenated tryptamine derivatives have been suggested as potential radiopharmaceuticals for monitoring abnormal brain states occurring in Parkinson’s and Alzheimer’s diseases and schizophrenia [50, 51] and tryptamine Schiff bases were proposed as antimicrobial agents [52] (Table 3). Tryptamine has also been targeted in plant biochemistry and biology, both as the precursor of the plant auxin IAA and as a proto-alkaloid in tobacco plant biology and of hallucinogenic derivatives.

## 5 The Transamination or Indolepyruvic Acid Pathway

### 5.1 General features and control

**Figure 3:**
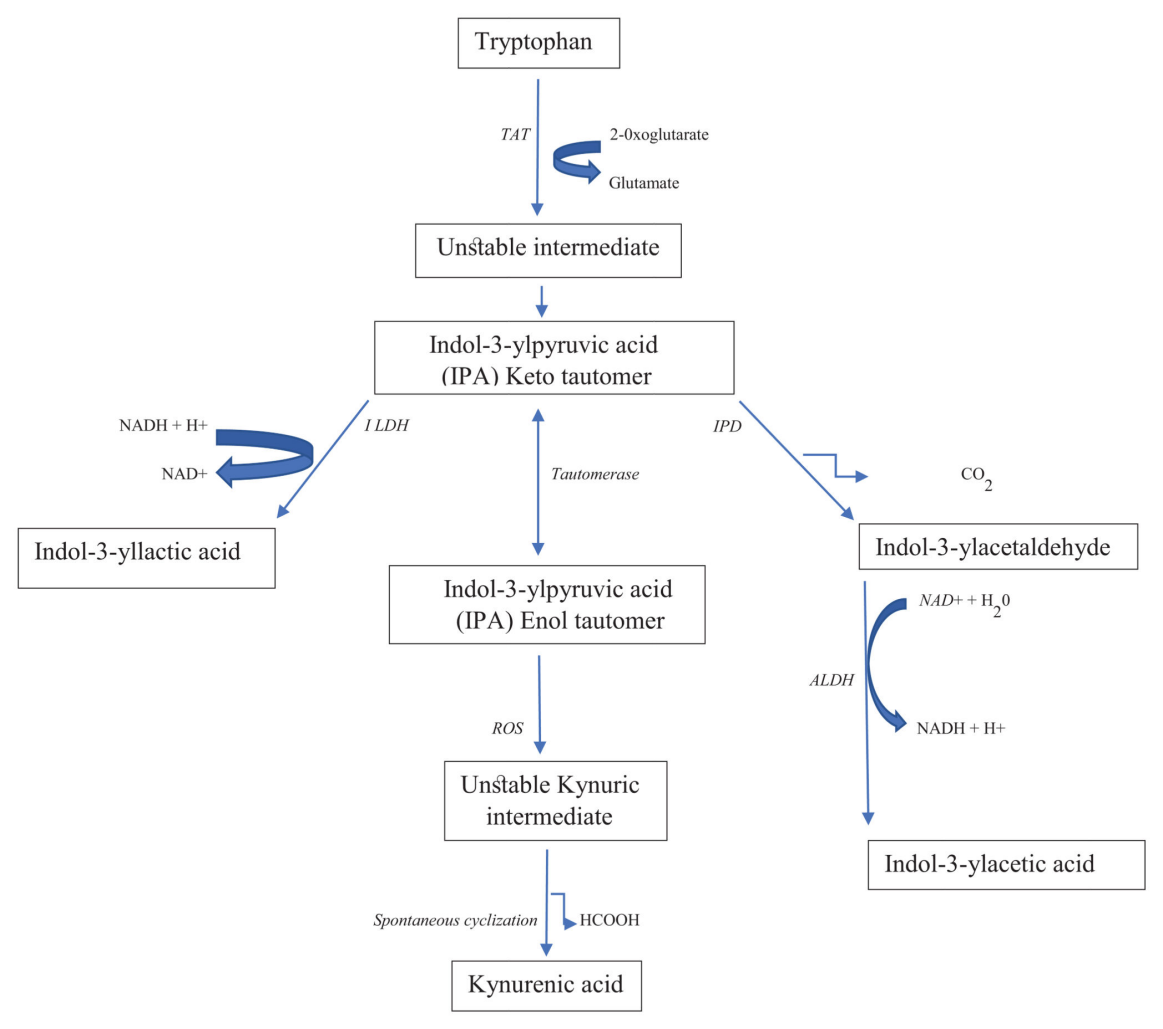
The transamination or indolepyruvic acid pathway. Abbreviations used are: ALDH (aldehyde dehydrogenase), IPD (indole pyruvate decarboxylase) ILDH (indole lactate dehydrogenase), ROS (reactive oxygen species), TAT (tryptophan aminotransferase).

Trp can be transaminated to indol-3-ylpyruvic acid (IPA) via an unstable intermediate (Figure 3) by aminotransferases. In crude homogenates of rat liver, 60% of Trp aminotransferase activity can be attributed to tyrosine aminotransferase [53]. Several more Trp-specific aminotransferases have now been identified. These include the mammalian/bacterial Trp-2-oxoglutarate aminotransferase (EC 2.6.1.27), which can also act on 5-HTP and to a lesser extent on phenyl amino acids, and the bacterial Trp-phenylpyruvate aminotransferase (EC 2.6.1.28) and plant Trp-pyruvate aminotransferase (EC 2.6.1.99) [54]. The IPA formed can be either reduced to indol-3-yllactic acid by indole lactate dehydrogenase, or decarboxylated by indole pyruvate decarboxylase to indol-3-ylacetaldehyde. The latter is subsequently oxidised by aldehyde dehydrogenase to indol-3-ylacetic acid.

IPA has been shown in more recent years to be converted to kynurenic acid (KA), an important kynurenine metabolite (see below). As shown in Figure 3, IPA is capable of tautomerization and when the keto form initially produced tautomerizes to the enol form, the latter is converted into an unstable Kynuric acid intermediate under the influence of reactive oxygen species (ROS), which spontaneously cyclises to KA [55]. Two potential mechanisms of KA formation from IPA are: non-enzymic production by ROS and production by transamination of kynurenine produced from Trp through IPA back transamination (see [55] and references cited therein).

It has been known for some time that IPA [56] is remarkably efficient in promoting the growth of rats deprived of Trp, thus suggesting that IPA can be back-transaminated to Trp. This could occur by the reversible transamination reaction of the mammalian enzyme [55], or of the common gut firmicute *Clostridium sporogenes* [57]. Synthesis of Trp from IPA was suggested in a study of a healthy woman who restored her nitrogen balance by intake of IPA while being maintained on a synthetic Trp-free diet [58].

As is the case with the decarboxylation pathway, the transamination or IPA pathway can be limited by availability of the PLP cofactor as determined by nutritional, pharmacological and physiological modulators.

### 5.2 Pharmacological targeting

IPA possesses a number of pharmacological properties, including sedation, analgesia, sleep-promotion and antioxidant and anticonvulsant actions [59, 60], which render it a target for pharmacological interventions (Table 3). A patent on “3-Indolepyruvic acid derivatives and pharmaceutical use thereof” for the treatment of disturbances of the central nervous system caused by elevation of brain superoxide anions in conditions such as epilepsy, cerebral ischemia, ictus and Alzheimer’s disease exists [61] which is hypothesized to involve increased production of KA from IPA. More recently, IPA has been shown to be formed and excreted in large amounts by the parasite *Trypanasoma brucei*, which causes African trypanosomiasis or sleeping sickness in humans and nagana in domestic animals [62]. IPA was shown to prevent the lipopolysaccharide (LPS or endotoxin)-induced glycolytic shift in macrophages resulting from increased hydroxylation and degradation of the transcription factor hypoxia-inducible factor-1*α* (HIF-1*α*). The reduction in HIF-1*α* levels by IPA following LPS or trypanosome activation results in a decrease in production of the proinflammatory cytokine IL-1β, thus permitting immune escape by the parasite (see the immune discussion in 6 below).

## 6 The Oxidative or Kynurenine Pathway

The KP exists mainly in the liver, where it accounts for ~ 90% of overall Trp degradation. The KP also exists in extrahepatic tissues and contributes little (< 2%) to Trp degradation under normal physiological conditions. However, after immune activation, the extrahepatic KP assumes a greater quantitative significance (see below). Whereas early studies of the KP were related mainly to its role in nutrition (exerted by the pellagra-preventing factor nicotinic acid or nicotinamide: the 2 forms of “vitamin” B_3_) and assessment of the functional capacity of the pathway by analysis of urinary metabolites following acute Trp loading in healthy subjects and those with various diseases, emphasis has shifted to assessment of the role of KP metabolites as modulators of immune function. Because of its special importance in health and disease, a detailed account is given below of the biochemistry and functions of the KP and the wide range of pharmacological targeting it attracts.

### 6.1 General features

**Figure 4:**
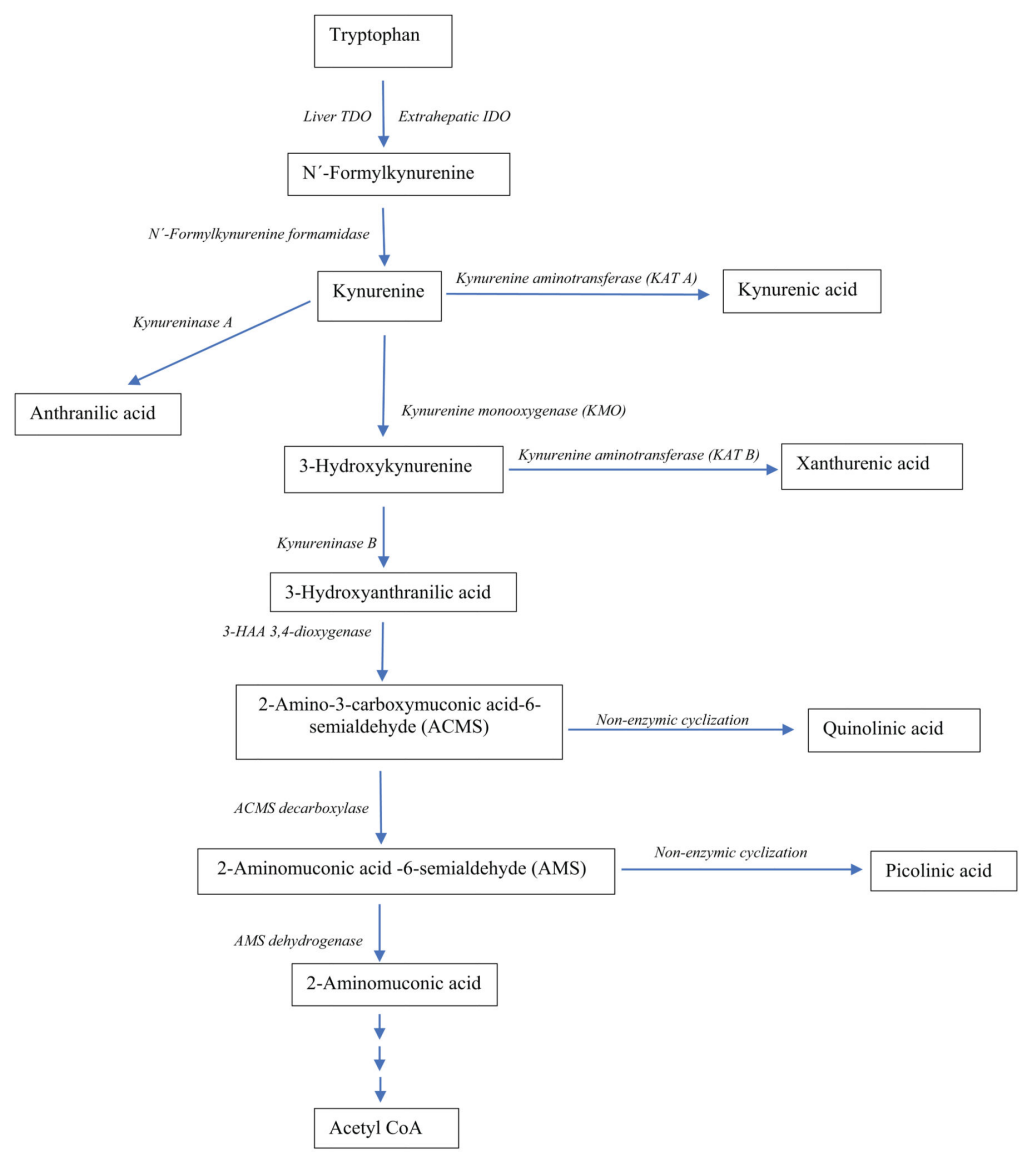
The oxidative or kynurenine pathway up to quinolinic acid and down to acetyl CoA. Adapted from Figure 1 in ref [6] (A. A.-B. Badawy. Kynurenine pathway of tryptophan metabolism: regulatory and functional aspects. Int J Tryptophan Res. 10, 1-20, 2017 doi: 10.1177/1178646917691938. Abbreviations used are: ACMS (2-Amino-3-carboxymuconic acid-6-semialdehyde), AMS (2-Aminomuconic acid -6-semialdehyde), IDO (indoleamine 2,3-dioxygenase), TDO (tryptophan 2,3-dioxygenase).

Detailed accounts of the KP have been published [6, 7]. As shown in Figure 4, Trp is first oxidized to N’-formylkynurenine by the catalytic actions of liver Trp 2,3-dioxygenase (TDO, formerly Trp pyrrolase; EC 1.13.11.11) and the extrahepatic indoleamine 2,3-dioxygenase (IDO: EC 1.13.11.17). The above product is rapidly hydrolysed by the abundant N’-formylkynurenine formamidase to kynurenine (Kyn). Kyn is then metabolised mainly by oxidation, first to 3-hydroxykynurenine (3-HK) by Kyn monooxygenase (KMO or Kyn hydroxylase), followed by hydrolysis of 3-HK to 3-hydroxyanthranilic acid (3-HAA) by kynureninase. This latter enzyme can also convert Kyn to anthranilic acid (AA). For simplicity, the kynureninase reactions will be designated as kynureninase A (Kyn → AA) and B (3-HK → 3-HAA). 3-HAA is then oxidized by 3-HAA 3,4-dioxygenase (3-HAAO) to the unstable intermediate 2-amino-3-carboxymuconic acid-6-semialdehyde (ACMS; also known as Acroleyl aminofumarate), which occupies a central position at the 2 arms of the KP. ACMS undergoes non-enzymic cyclization to quinolinic acid (QA) or decarboxylation by ACMS Decarboxylase (ACMSD: also known as picolinate carboxylase) to 2-aminomuconic acid-6-semialdehyde (AMS). The latter can also undergo non-enzymic cyclization to picolinic acid (PA) or oxidation by AMS dehydrogenase to 2-aminomuconic acid, with eventual conversion to acetyl CoA. The KP favours the production of QA rather than PA. Only when AMSD is saturated with its substrate can PA be formed in larger amounts. The KP includes 2 aminotransferases, which transaminate Kyn to KA (KAT A) and 3-HK to xanthurenic acid (XA) (KAT B). Transamination is, however, a minor pathway, in view of the high K_*m*_ of the 2 enzymes for their substrates (Kyn and 3-HK), compared with the lower K_*m*_s of KMO and kynureninase for these 2 substrates respectively (see [6] for details). Only when the substrate concentration is increased [by Trp or Kyn loading or KMO inhibition (see 6.4.6. below)] can significant amounts of products be formed. As will be described below, 4 isoforms of KAT are known (KAT I, II, III and IV).

**Figure 5:**
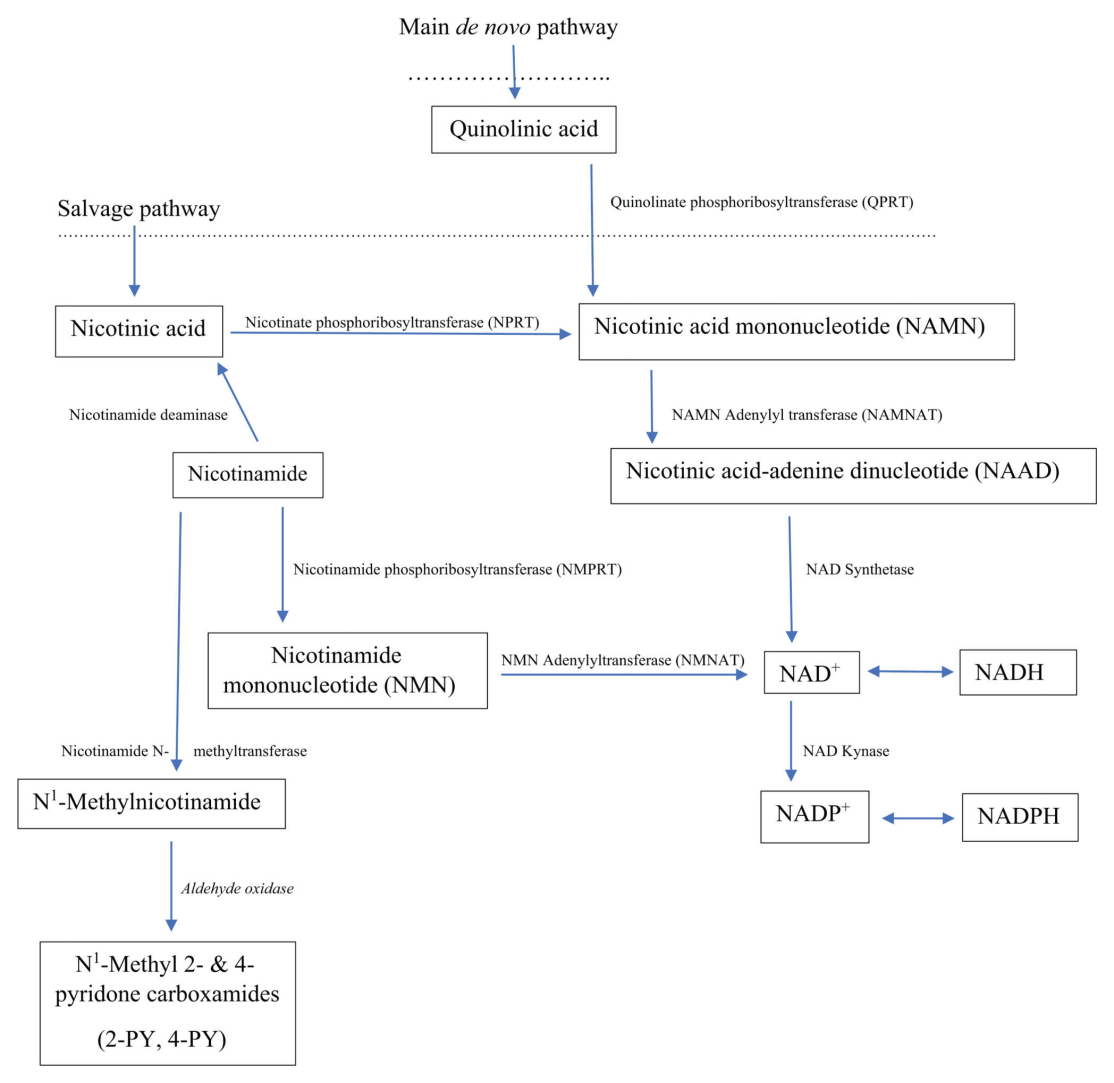
NAD^+^ synthesis from quinolinic acid and via the salvage pathway. Adapted from Figure 1 in ref [6] (A. A.-B. Badawy. Kynurenine pathway of tryptophan metabolism: regulatory and functional aspects. Int J Tryptophan Res. 10, 1-20, 2017 doi: 10.1177/1178646917691938.

The rest of the KP involves the synthesis of NAD^+^ from QA (the *de novo* pathway), or from nicotinic acid and nicotinamide (the salvage pathway) through the series of reactions outlined in Figure 5, which also shows the interconversion of NAD^+^ and NADP^+^ and the methylation of nicotinamide and subsequent oxidation to the major urinary metabolites the N^1^ methyl 2- and 4-pyridone carboxamides. Details of all KP enzymes, including substrates, products, cofactors and major tissue sources can be seen in [6]. The liver is the only tissue that contains the complete set of enzymes of the KP leading to niacin and NAD^+^ synthesis. Thus, in extrahepatic tissues, the presence or absence of certain enzymes will determine the KP metabolite patterns in these tissues. NAD^+^ synthesis is more effective from Trp (via QA), than from nicotinamide or nicotinic acid [63–65]. From a nutritional viewpoint, a 1 mg of niacin is formed from 60 mg of Trp [66, 67].

### 6.2 Control of the pathway

The KP is controlled primarily by the first enzyme(s), namely TDO in liver and IDO elsewhere. Although these are the 2 rate-limiting enzymes of the KP, it is important to emphasize that the flux of Trp down the pathway is determined primarily by plasma free Trp (see [6] and references cited therein). More recent evidence (see below) suggests that some Kyn metabolites can also play a regulatory role within the pathway.

#### 6.2.1 Roles of TDO and IDO in tryptophan oxidation

As stated above, liver TDO controls Trp oxidation through the KP under normal physiological conditions, whereas the extrahepatic IDO plays a negligible role. Evidence for this difference is provided by the observations that deletion of the TDO2 gene increases plasma [Trp] by 9.3-12.7-fold [68, 69] and that, although plasma [Trp] was not measured in a study involving IDO1 deletion [70], brain [Trp] was not altered after IDO1 or IDO2 gene deletion in contrast to a 10.6-fold increase in brain [Trp] after TDO2 gene deletion [71]. However, as will be discussed below, under conditions of immune activation, IDO assumes a greater role in Trp oxidation, with important biological consequences. Under certain conditions, the KP may also be limited by activity of certain enzymes, notably kynureninase and ACMSD or picolinate carboxylase.

#### 6.2.2 Regulation of TDO

Liver TDO in humans, rats and certain, but not all other, animal species exists in two forms: the active haem-containing holoenzyme and the inactive haem-free apoenzyme, in roughly equal proportions. Other differences between these two groups of animal species exist, which necessitate careful choice of the most suitable animal model to study in relation to human Trp metabolism. This will be discussed below.

In rats, TDO is regulated by 4 mechanisms: (1) glucocorticoid induction of *de novo* synthesis of the enzyme; (2) substrate (Trp) activation of the enzyme by promoting the conjugation of the haem-free apoenzyme with its haem cofactor and by stabilisation of the pre-existing apoenzyme in the presence of the normal rate of its synthesis; (3) cofactor activation by haem; (4) feedback inhibition by NAD(P)H. TDO was one of the first enzymes in which these mechanisms were identified, which established the concept of enzyme regulation in the early 1960s, and this was pioneered in the USA by the groups of W Eugene Knox, Olga Greengard, Philip Feigelson and Henry C Pitot. Details of these mechanisms are discussed in [6] and it is noteworthy that: (1) some other hormones influence TDO synthesis; (2) the Trp activation of TDO may involve induction of haem biosynthesis; (3) the haem cofactor may mediate glucocorticoid induction of TDO mRNA transcription and translation; and (4) regulate the TDO gene post-translationally through enhanced phosphorylation of the *α* subunit of the eukaryotic initiation factor eIF2*α* (for references, see [6]). Regarding the feedback allosteric inhibition of TDO [72], the enzyme activity is inhibited by agents which increase the hepatic concentrations of NAD(P)H, such as glucose and nicotinamide [73] and chronic ethanol intake [74].

#### 6.2.3 Regulation of IDO

By contrast with liver TDO, the extrahepatic IDO is not inducible by glucocorticoids nor activated by haem, as it exists only in the haem-containing active holoenzyme [75, 76]. Also, IDO is less sensitive to activation by Trp, compared with TDO. Thus, whereas the latter in rat liver is activated severalfold by Trp, IDO in the rat intestine (the richest source) is activated by only 50% by a large Trp dose [76]. This modest response may involve substrate inhibition of IDO, which occurs with the mouse epididymal enzyme at [Trp] above 50 *μ*M [77] by a mechanism investigated in the human enzyme involving a reversed sequence of binding of Trp and O_2_ [78]. At low [Trp], Trp is bound to the enzyme first followed by O_2_, whereas at high [Trp], this order is reversed, with the haem reduction potential playing an important role [78].

The principal effector of IDO is interferon-*γ* (IFN-*γ*) [79]. Interferon-*α* is a less efficient inducer [80]. Other cytokines and mediators (both pro- and anti-inflammatory) exert various effects on IDO (for details and references, see [34]). Thus, the IDO status can be assumed to be determined by the balance between pro- and anti-inflammatory cytokines. Potentiation of the IFN-*γ* induction of IDO by the synthetic glucocorticoid dexamethasone, which is ineffective by itself [80] suggests that glucocorticoids exert a permissive effect on IDO induction, which may be important in tumoral immune escape (see below).

As an inhibitor of human IDO [81], nitric oxide (NO) may act as modulator of the immune function of the enzyme. Activity of the recombinant human IDO is reversibly inhibited by NO by binding to haem, with the inactivated enzyme complex being the Fe^2+^-NO-Trp adduct [82].

#### 6.2.4 Species differences in tryptophan metabolism and the choice of animal models

Because of wide species differences in Trp metabolism and enzymes of the KP, it is important that the appropriate animal model of Trp-related function and in relation to human disease is carefully selected. Regarding Trp metabolism, species can be broadly divided into 2 groups: one possessing both forms of TDO (holoenzyme and apoenzyme) and the glucocorticoid induction mechanism, and the other lacking the free apoenzyme and glucocorticoid induction. The former group includes man, rat, mouse, chicken, turkey and pig, whereas the second group includes cat (*Felis catus*), frog, the Mongolian gerbil, the golden (Syrian) hamster, guinea pig, ox, rabbit and sheep [83]. Because of the absence of the glucocorticoid induction mechanism, which facilitates increased TDO synthesis to handle a sudden increase in [Trp], the latter group of species are sensitive to the toxicity of excess Trp [83]. There are also major species differences in enzymes of the KP (other than TDO) in liver and elsewhere among the above 2 groups. A most notable difference between cat and rat is the much greater activity of ACMSD, with a cat:rat ratio of 32: 1 in liver and 4: 1 in kidney [84]. This greater ACMSD activity renders the cat vulnerable to niacin and NAD^+^ deficiency, with the conversion of 3-HK to niacin ribonucleotides being only 11% of that in the rat [84]. More recently, differences in many KP enzymes in various tissues have been reported between rats, mice, rabbits, gerbils and guinea pigs [85, 86]. The distinction between these 2 groups of species based on differences in TDO and other KP enzymes does not apply to IDO [85, 86].

It may therefore be concluded that the above TDO-deficient species are unsuitable as animal models of human Trp-related diseases, though they would be valid models in studies addressing issues related to their specific KP characteristics. Rats and mice are the most common experimental animal models. However, because of significant mouse strain differences in Trp metabolism [12], I recommend the Wistar rat as the most suitable animal model for Trp-related studies.

### 6.3 Functions of the pathway

The KP performs a variety of important physiological functions, disturbances of which result in negative health consequences. The following are brief accounts of these functions, (Table 4), which have been discussed in more detail elsewhere [6].

#### 6.3.1 Detoxification of excess tryptophan

As stated above, animal species lacking the free TDO apoenzyme and its glucocorticoid induction mechanism are sensitive to the toxicity of excess Trp [83]. These species are unable to synthesise the TDO apoenzyme necessary to meet the increased need to process the excess Trp to “harmless” kynurenine metabolites. As a result, Trp metabolism is diverted towards production of excessive amounts of indoles [87]. When rats are deprived of the glucocorticoid induction mechanism by adrenalectomy, they become also sensitive to the toxic actions of excess Trp but receive protection upon cortisol administration [88]. This detoxicating function of the KP is restricted to liver TDO and so does not involve differences in IDO, because, although some have a higher IDO activity, species lacking the TDO apoenzyme are still sensitive to Trp toxicity. The ability of [Trp] above 50 *μ*M to inhibit IDO activity [77] may make a possible additional, if minor, contribution to the poor ability of these species to process the excess Trp. It is notable that, with the exception of the cat, these species are herbivorous. They, however, attempt to deal with Trp differently from species which tolerate Trp, by two mechanisms. First, their TDO shows a rapid response to activation by Trp and at lower [Trp], compared with e.g. rats (see [89] and references cited therein). Second, they metabolise Trp to acetyl CoA more efficiently than the other species. Thus, as described above, the cat possesses a greatly elevated ACMSD activity [84] and a study in isolated hepatocytes [90] showed that, whereas the rate of Trp oxidation and QA formation are lower than in rats, guinea pigs, gerbils and sheep metabolize a much larger part of Trp through the citric acid cycle.

#### 6.3.2 Control of plasma tryptophan availability

As stated in 6.2.1. above, TDO controls plasma Trp availability under normal physiological conditions, whereas IDO plays a more active role during immune activation. Earlier work has demonstrated the impact of liver TDO on plasma Trp availability. For example, an inverse relationship exists between liver TDO activity and brain [Trp] and 5-HT synthesis [91]. The potential glucocorticoid induction of TDO by the elevated cortisol may explain the serotonin deficiency in major depressive disorder (MDD) [34]. In this latter study, it was shown that TDO inhibition by chronic administration to rats of drugs of dependence increases brain [Trp] and enhances 5-HT synthesis, whereas TDO induction by corticosterone during subsequent drug withdrawal exerts the opposite effects. The dramatic increase in plasma [Trp] induced by deletion of the mouse TDO gene results in an equally dramatic increase in circulating Trp availability to the brain (expressed as the [Trp]/[competing amino acids] ratio) and consequently in brain Trp, 5-HT and 5-HIAA [68]. The control of Trp availability to the brain by TDO has also been suggested [68] as a modulator of anxiety through changes in brain 5-HT. TDO gene deletion also increases Trp availability for Trp decarboxylation and transamination [68] and it is of interest that the Trp transamination product indolepyruvic acid (IPA) possesses anxiolytic properties [60].

Although it plays a minor role in the control of plasma Trp availability under normal conditions, the extrahepatic IDO can influence KP activity even in the absence of TDO, as suggested by the finding [68] that plasma [Kyn] and [KA] are maintained at wild-type levels in TDO Knock-out (KO) mice. In the absence of preformed niacin, synthesis of Kyn and its metabolites KA, XA and 3-HAA is increased, whereas that of QA is decreased, in these mice [69]. The latter authors suggested that Kyn formed by IDO extrahepatically can be utilized by the liver to form adequate amounts of nicotinamide and NAD^+^ nucleotides to maintain growth. Also, it is very likely that the hepatic KP can contribute to extrahepatic Kyn metabolite formation through the availability of the Kyn precursor. In some situations, this is likely to be quantitatively more important than the modest contribution of IDO itself. The role of IDO in control of plasma Trp availability takes centre stage when the immune system is activated. IDO induction by IFN-*γ* and agents acting through it leads to depletion of [Trp] and increased Kyn formation in cultures of monocytes [80] and serum [92].

#### 6.3.3 Control of hepatic haem biosynthesis

Mammalian hepatic haem biosynthesis is controlled by the rate-limiting enzyme 5-aminolaevulinate synthase (5-ALAS) and is achieved by a negative feedback mechanism exerted by a small pool of haem whose concentration in the (rat) hepatic cytosol is ~ 10^−7^ M (see [93] and references cited therein). Haem biosynthesis can therefore be enhanced by a decrease in this regulatory-haem pool, e.g. by destruction of haem to green and other pigments by chemical porphyrogens, such as 2-allyl-2-isopropylacetamide and 3,5-diethoxycarbonyl, 1,4-dihydrocollidine), inhibition of ferrochelatase by griseofulvin, or induction of haem oxygenase by metal cations. Under any of these conditions, the decrease in the regulatory-haem pool removes the negative feedback control. Utilisation of this pool by haemoproteins is another mechanism by which haem biosynthesis can be enhanced. The only haemoprotein that utilises this pool is liver TDO [93]. This utilisation can be estimated from the increase in the TDO holoenzyme activity or in the haem saturation of the enzyme in rat liver. The latter is usually expressed as the percentage haem saturation (100 X holoenzyme activity/total enzyme activity) or the haem-saturation ratio (holoenzyme activity/apoenzyme activity) [93]. Under a variety of experimental conditions involving changes in haem synthesis and degradation, the above haem saturation of TDO is altered in the appropriate direction and is always inversely related to 5-ALAS activity [93–97]. The rat (and possibly also human) liver TDO thus serves as a sensitive marker of changes in the regulatory-haem pool: a property that forms the basis of a screening test for exacerbation of porphyria by drugs and other chemicals [98].

#### 6.3.4 Modulation of immune function by kynurenine metabolites

The first indication that Trp metabolites along the KP may influence immune function was the discovery in the 1970s-80s of the enzyme indoleamine 2,3-dioxygenase (IDO) and its induction by interferon-*γ* (IFN-*γ*) [99–101]. Initially, it was thought that the antibacterial, antiparasitic and antiviral effects of this major cytokine involve deprivation of these pathogens of an essential nutrient, Trp, by stimulating its breakdown through IDO induction [102, 103]. The Trp depletion theory in infectious diseases was thus born and its application was extended to explain the immune tolerance of pregnancy [104]. However, Trp is not depleted in pregnancy, but is increased to meet the increased demand for protein synthesis by mother and foetus. The above decrease refers to the plasma total [Trp], which has been shown in many studies to occur in late pregnancy. However, in contrast, maternal free [Trp] is increased throughout pregnancy by a combination of liver TDO inhibition, increased [NEFA] and decreased [albumin, [105]]. This illustrates the need to measure both free and total [Trp] for accurate interpretation of changes in Trp metabolism and disposition. While there may be a case for Trp depletion in infection, this cannot be a universal or a sole mechanism for defence against pathogens. For example, most bacteria will not suffer from Trp depletion, because they can synthesize Trp. Several other equally compelling arguments against the Trp depletion concept can be seen in the excellent review by Moffett and Namboodiri [106]. These latter authors proposed “Trp utilisation” as a more appropriate concept in infectious diseases. A Trp utilisation concept in pregnancy was also proposed [107, 108]. The Trp depletion concept in pregnancy and infection has been suggested [109] to be no longer tenable, because the depletion of Trp is accompanied by increased formation of kynurenine metabolites, which exert profound effects on the immune system. The Trp utilisation concept was thus born and the following account summarises the immunomodulatory properties of kynurenine metabolites.

QA was the first Kyn metabolite to be shown to possess antiinflammatory effects in rats [110]. Subsequent studies showed that other Kyn metabolites (Kyn, 3-HK and 3-HAA) suppress T cell responses *in vitro* in an additive manner and by an apoptotic mechanism [111]. Findings by Fallarino *et al* [112] confirmed the apoptotic mechanism and showed that both QA and 3-HAA induced apoptosis in T-helper type 1 (Th1), but not in Th2 cells. At the smallest concentration of Kyn metabolites tested (10 *μ*M), only 3-HAA and QA induced apoptosis in thymocytes, whereas 3-HK, Kyn and AA were ineffective. With macrophages, a 10-fold higher concentration of 3-HAA was needed to induce apoptosis. Other details are discussed in [106] and further information on the immunomodulatory effects of Kyn metabolites continues to be generated by many subsequent and current investigations. As will be discussed below, tumors take advantage of the immunosuppressive effects of Kyn metabolites to undermine effector T cell function and thereby escape an immune response.

The immunomodulatory effects of Kyn metabolites have, however, been demonstrated *in vitro* at concentrations (10 *μ*M and above) that are much higher than their circulating plasma levels, which are in the sub-micromolar range (see [113] and references cited therein). Metabolites could, however, reach very high levels in cellular microenvironments. Attempts to establish such levels have been successful only with QA using immunohistochemistry [106, 114]. This has been possible by the successful production of specific antibodies to QA by virtue of its chemical structure, in particular the absence of an amino group. With other Kyn metabolites, except for KA and PA, the presence of both an amino and a carboxyl group allowed orientation in several directions when being coupled to a protein (see [113]), thus leading to non-specific epitopes. However, with 3-HAA, it is possible that much of it may be converted to QA within microenvironments, because 3-HAA 3,4-dioxygenase is the most active enzyme of the KP [115, 116]. However, for practical reasons and until suitable methods are devised to measure other Kyn metabolites in cellular microenvironments, emphasis should be placed on QA measurements.

Traditionally, studies of QA and KA have been conducted in relation to their actions at the NMDA (N-methyl-D-aspartate) type of receptors of the excitatory amino acid glutamate following the pioneering discovery by T W Stone [117] of QA as agonist and KA as antagonist at these receptors. This opened a new field of investigation of Trp metabolism in cognitive and neurological diseases, with QA being neuronal excitotoxic and KA being cytoprotective. While QA can modulate the immune system to induce T cell suppression, and thus acts as a pro-inflammatory Trp metabolite, KA possesses antiinflammatory properties (see [113] and references cited therein). KA has so far received only minimal attention in studies on inflammatory diseases and growing evidence now suggests that it should receive greater emphasis in future studies. It would therefore appear that, as in the case of cognitive and neurological diseases, QA and KA may also play opposing roles in inflammatory diseases [113].

In the above hypothesis [113], the potential role of anthranilic acid (AA) in inflammation is discussed. AA is not without immunomodulatory activity and many anti-inflammatory drugs have been developed from the AA nucleus, including mefenamic acid and diclofenac. The 5 hydroxylated AA metabolite (5-HAA) is a potent apoptotic agent with a potency equal to that of 3-HK and 3-HAA (see [113] and references cited therein). Darlington et al [118] reported a decrease in the ratio in plasma of [3-HAA]/[AA] in a variety of neurological and neurodegenerative diseases. This decrease is due to a rise in [AA] and in some cases also a decrease in [3-HAA]. The authors [118] suggested that this decrease either reflects inflammatory disease and its progression or is an antiinflammatory response. I hypothesized in favour of the latter possibility [113] based among others on the potential role of KA in the AA elevation. We have previously reported [119] that KA administration to rats increases liver [AA] by stimulating the kynureninase A reaction (Kyn → AA). In this latter study [119], various changes in KP enzyme activities were observed following administration of Kyn metabolites. Notably: (1) KA stimulated TDO possibly by acting via 3-HAA, but inhibited KAT activity; (2) 3-HK inhibited the kynureninase B reaction (3-HK → 3-HAA); (3) 3-HAA stimulated TDO but inhibited the kynureninase A and B reactions. These novel effects of Kyn metabolites point towards new and hitherto unrecognised internal mechanisms of control of the KP by its intermediates that may contribute to the overall activity of the pathway.

#### 6.3.5 Modulation of carbohydrate metabolism and other processes by kynurenine metabolites

Carbohydrate metabolism and its impact on diabetes can be influenced by kynurenine metabolites, notably QA, XA and PA. For example, activity of the key gluconeogenic enzyme phospho-enol-pyruvate carboxykinase is inhibited by QA. In species in which Trp conversion to QA is strong, e.g. the rat, gluconeogenesis is inhibited by Trp administration, whereas this is not the case in species, such as gerbil, guinea pig or sheep, which exhibit poor QA production from Trp [90, 120, 121]. Because of KP similarities with rats, gluconeogenesis is likely to be inhibited in humans under conditions of excessive QA elevation or production. It is noteworthy that animal models of diabetes are mainly those of rodents and pigs, but not species in which QA production is limited.

Carbohydrate metabolism could also be influenced by XA and PA acting on insulin. The diabetogenic effect of XA is thought to involve binding of and hence inactivating insulin [7]. A high plasma [XA] is associated with high insulin resistance and higher odds of having diabetes [122]. Urinary and plasma [XA] is elevated in diabetic patients and experimentally-induced diabetes in rats [123, 124]. The increased urinary XA excretion is accompanied by that of Zn in the form of an XA-Zn complex [123]. Insulin requires Zn at many levels [125]. Zn absorption and bioavailability are, however, controlled by another Kyn metabolite picolinic acid (PA). While there are no available data on [PA] in diabetes, current evidence suggests a likely increase. Thus, ACMSD activity and mRNA expression are increased in experimental diabetes, although hepatocyte PA production is not impaired, and ACMSD activity is greater in kidney than in liver (for references, see [6]). Plasma [PA] is however elevated in hepatitis C viral infection, and to a greater extent if diabetes is present [126]. From this account, it appears that further work on the roles of XA and PA in diabetes is required, e.g. to establish if the increased urinary excretion of Zn in diabetes is due in part to a potential defect in binding to PA in addition to complex formation with XA.

PA and XA are the least studied metabolites of the KP. PA exerts immunomodulatory effects (see [109]) and its levels are increased in plasma in hepatitis C viral infection and hepatic cirrhosis [126] and in CSF in cerebral malaria [127]. Little else is known about the status of this KP metabolite in other CNS conditions [128]. With XA, other than its insulin and Zn binding described above and activation of the malaria gametocyte [129], evidence exists for its involvement in synaptic signaling and hence neurotransmission [130]. Thus, more work on these 2 KP metabolites is required. Kyn metabolites also act as ligands of the aryl hydrocarbon receptor (AhR), the significance of which will be described in 6.4.2. below.

Kyn metabolites may also play important roles in conditions not directly associated with inflammation. These include inhibition by KA of alcohol- and cocaine-seeking behaviour and relapse and induction of aversion to alcohol by KA, 3-HK and 3-HAA (for references, see [113, 119]).

#### 6.3.6 Niacin synthesis and pellagra prevention

In the absence of adequate intake of niacin (in the form of nicotinic acid or nicotinamide), its levels are maintained by *de novo* synthesis from Trp via the QA arm of the pathway. Niacin deficiency is the central feature of pellagra, usually referred to as the disease of the 3 Ds (dermatitis, diarrhoea and dementia, though more appropriately delirium). Tissues with a great demand for nicotinamide-adenine dinucleotides to meet their rapid cellular turnover, e.g. skin, gastrointestinal tract and the nervous system, suffer most from the resultant NAD^+^ deficiency.

Nutritional pellagra therefore occurs only if diets are deficient in both niacin and Trp. Even with marginal niacin, but adequate Trp, intake, clinical or subclinical pellagra can be induced by drugs interfering with one or more enzymes of the KP, e.g. by TDO inhibition by some antibiotics and antiviral drug or kynureninase inhibition by hydrazine compounds or oestrogens [7, 131].

Subsistence on a largely maize staple [7, 131] led to a widespread incidence of pellagra in Southern Europe during the 18th century and in the USA following the American civil war. Although maize (and sorghum, widely used in India) are Trp-deficient, they contain adequate amounts of niacin, but in a polysaccharide-bound form (niacytin) that cannot be hydrolysed by mammalian digestive enzymes. Unfortunately, those who introduced maize in Southern Europe ignored the importance of the liming process, a procedure used for millennia by the peasants of Central America in the preparation of tortillas, that causes the release of niacin from niacytin [131]. Also, the presence of high levels of leucine in maize and sorghum aggravates the pellagra by activation of TDO and ACMSD and inhibition of kynureninase and QPRT [7, 131, 132]. While pellagra continues to result from malnutrition in certain parts of the world, it appears occasionally in developed countries in association with alcoholism and it may be relevant that, among other effects, chronic ethanol consumption inhibits TDO activity [74, 132].

It is generally accepted that 1 mg of niacin arises from intake of 60 mg of Trp [66, 67], though this ratio shows individual variations and can be influenced by factors including nutrients, hormones, pregnancy, drugs and diseases, with some nutrients and hormones enhancing and others suppressing the conversion of Trp to nicotinamide [133]. The niacin status is generally determined by measuring urinary excretion of N^1^-methylnicotinamide and its 2 oxidation products 2-PY and 4-PY (Figure 5) [134, 135]. The correlations between daily niacin intake and urinary excretion of 2-PY and 4-PY are comparable and more superior to that between niacin intake and N^1^-methylnicotinamide excretion [135]. TDO gene deletion in mice, however, still allows synthesis of nicotinamide and NAD^+^ to proceed [69].

#### 6.3.7 Control of NAD^+^ synthesis

The final major function of the hepatic KP is production of the redox cofactor NAD^+^, from which NADP^+^ is formed by the action of NAD^+^ kinase (Figure 5). Both oxidized dinucleotides and their reduced forms play vital roles in metabolism at multiple levels and in other cell functions and are therefore essential to life. As will be outlined in 6.4.10. below, defects in NAD^+^ availability have negative health consequences. As the KP favours the arm leading to QA formation (Figures 4 and 5), it must be concluded that NAD^+^ synthesis from QA and hence from Trp is quantitatively more important than that from nicotinamide or nicotinic acid. This is illustrated by the findings [63–65] that dietary Trp is more effective than dietary nicotinamide or nicotinic acid in elevating liver nicotinamide dinucleotides and urinary levels of N^1^-methylnicotinamide. Studies by the group of Bender [7, 64, 65] suggest that: (1) activities of nicotinamide deamidase (NMD) and nicotinamide phosphoribosyltransferase (NMPRT), both of which are substrate-saturated at normal (steady-state) levels of liver nicotinamide determine the incorporation of nicotinamide into the dinucleotides; (2) whereas activities of the above 2 enzymes show a significant correlation with hepatic nicotinamide dinucleotide levels, this is not case with nicotinic acid phosphoribosyltransferase (NPRT), which functions normally just below its V*_max_*; (3) by contrast, although QPRT activity also does not correlate with liver dinucleotides, this enzyme operates at [QA] well below its K_*m*_, thus suggesting that increased availability of QA could lead to greater formation of NaMN and hence NAD^+^; (4) although liver dinucleotide levels are increased after a single large dose of nicotinamide, this is more likely to result from decreased NAD^+^ catabolism, rather than increased synthesis, because both nicotinamide and its N^1^-methyl metabolite inhibit the NAD^+^-degrading (depleting) enzyme poly-(ADP-ribose) polymerase (PARP; EC 2.4.2.30). The significance of PARP will be discussed below.

It is important to note that, whereas QA does not accumulate in liver after Trp loading, presumably because of its rapid metabolism to NAD^+^, activated cells of the immune system accumulate relatively large amounts of QA and it has been suggested that this is to provide the substrate for NAD^+^ synthesis and the PARP reaction in response to immune-related oxidative damage [106].

### 6.4 Pharmacological targeting of the pathway

The relatively large number of enzymatic steps in the KP renders it open to multiple targeting for pharmacological intervention. Of the actual precursors and intermediates of the pathway, Trp, nicotinic acid, nicotinamide and kynurenic acid can also be targeted for therapeutic use. The Trp literature includes details of research on and application of, individual targets, and only brief accounts will therefore be presented in this section.

#### 6.4.1 Tryptophan, nicotinic acid and nicotinamide

As stated in 3.2, Trp has been used as antidepressant, with only modest efficacy when used alone, but more effectively in combination with antidepressants [34]. Many antidepressants also inhibit liver TDO activity and lower circulating cortisol, thus providing the means of preventing excessive hepatic Trp degradation, thereby increasing Trp availability to the brain for 5-HT synthesis. Potent TDO inhibitors have been developed for use in cancer immunotherapy (see below) and there is no reason why they should not be effective as antidepressants, either alone or in combination with Trp. As will be discussed below, the same principle applies to the potential treatment of acute hepatic porphyrias by TDO inhibitors.

Targeting Trp availability to tumors has been suggested as a strategy to overcome tumoral immune escape [13]. Tumors need Trp and other nutrients to stimulate their growth and proliferation. They upregulate amino acid transporters, 4 of which (SLC1A5, SLC7A5, SLC7A11 and SLC6A14) are of special interest in cancer biology [136]. Of these, SLC6A14 transports all essential amino acids [137]. *α*-Methyltryptophan (*α*-MT) inhibits SLC6A14 function thereby preventing amino acid uptake by cancer cells [138] and has been shown to undermine growth of oestrogen receptor-positive breast cancer cells [139]. Tumors are sensitive to changes in [Trp]. When [Trp] is decreased to ≤ 5 *μ*M, tumors upregulate specific Trp transporters [140]. At the same time, the increased uptake of Trp coupled with upregulation of IDO and, where appropriate, TDO, ensures adequate formation of immunosuppressive Kyn metabolites (3-HK, 3-HAA, QA), which tumors use to undermine effector T cell proliferation and function and thereby escape an immune attack. T cell proliferation is inhibited at [Trp] of < 10 *μ*M [141]. Thus, the narrow range of [Trp] of 5–10 *μ*M is critical for survival of tumors and effector T cells. Consequently, strategies aimed at maintaining effector T cell function by ensuring that tumoral [Trp] remains above 10 *μ*M should be pursued in conjunction with strategies preventing production of immunosuppressive kynurenines, namely those involving inhibition of IDO/TDO upregulation and limiting Trp availability to tumors. In human colon and stomach cancer tissues, tumoral [Trp] can reach 70 and 40 *μ*M respectively and a much higher value (270 *μ*M) has been reported in a mouse model of CT26 colon carcinoma (for references, see [13]). In the above proposed targeting strategy [13], inhibition of amino acid transporter function by *α*-MT is the first line of action. Decreasing plasma free Trp availability to tumors, which is enhanced in cancer through decreased albumin and increased NEFA, could be achieved by albumin infusions and use of antilipolytic agents, e.g. nicotinic acid. IDO/ TDO inhibition should complete the proposed strategy and is discussed further below. Both nicotinic acid and nicotinamide have been used in therapy of certain cancers, but for reasons other than modulation of Trp metabolism and disposition (for references, see [13]). TDO inhibition by nicotinamide [73] will be discussed further below.

#### 6.4.2 Kynurenic acid

KA exerts effects in the central nervous system (CNS) and the periphery which render it a target for pharmacotherapy at various levels. In the CNS, blocking the excessive production of KA in schizophrenia requires the use of KAT inhibitors (see below). In the periphery, KA affects the immune system and gastrointestinal tract (GIT) function. It acts as a ligand for the orphan G protein-coupled receptor GPR35, which is expressed in both types of cells [142], but involvement of this receptor in the antiinflammatory activity of KA is somewhat controversial [142, 143]. The KA inhibition of tumour necrosis factor-*α* (TNF-*α*) production by mononuclear cells, high mobility group box protein 1 (HMGB1) production by monocytes and human neutrophil peptide 1-3 (HNP1-3) secretion by neutrophils has been shown [144] to be stronger with 2-(2-N,Ndimethylaminoethylamine-1-carbonyl)-1H-quinolin-4-one hydrochloride, a KA analogue, and exploration of this analogue activity in human inflammatory disease has been proposed. KA production and KAT activity are decreased in retinal ganglion cell loss during retinal neurodegeneration [145]. In a mouse model of ocular hypertension, age-related decreases in [KA] and KAT activities are observed [145]. Intense KAT activities have also been demonstrated in the corpora amylacea of the human retina [145]. These changes justify exploring a potential role of KA in these retinal conditions.

[KA] is relatively high in the GIT [146] and its serum concentration is elevated in inflammatory bowel disease [147], but is decreased in the non-inflammatory bowel condition, the irritable bowel syndrome [148, 149]. This latter study also showed decreased serum levels of Kyn and 3-HAA, suggesting inhibition of Trp degradation at a step(s) beyond that catalysed by TDO/IDO and it was also suggested that the reported increase in serum free [Trp] increases serotonin levels, which may explain the increased gut secretions and motility described in diarrhoea-predominant IBS.

Another receptor involving KA is the aryl hydrocarbon receptor (AhR), which is a ligand-activated transcription factor mediating the toxicity of environmental chemicals, such as the dioxins. Activation of the AhR induces toxic responses including cell damage and carcinogenesis [150]. The AhR can also control immune responses in both protective and destructive ways [150, 151]. For example, whereas endogenous ligands of the AhR facilitate a dampening of the immune response to prevent excessive inflammation and autoimmunity, exogenous ligands act as signals to enhance inflammatory responses to infection and resistance of cancer to its own destruction [150], resulting in a state of “pathological immunosuppression”, a mechanism of which based on changes in Trp availability has been suggested (see [6, 109]). Of the Trp metabolite ligands of the AhR (Kyn, KA and XA), KA has the highest ligand activity (see [109]). The dual role of activation of the AhR is illustrated by 2 examples involving KA. While KA activation of the AhR allows certain tumor cells to escape immune surveillance by secreting large amounts of IL-6 [152], deletion of the mouse AhR gene increases production of KA and expression of Kyn aminotransferase II (KAT II) in mouse cortex and striatum, thus protecting the brain against excitotoxicity and oxidative stress [153]. Other examples of this dual role and KA involvement are detailed in the excellent review by Wirthgen et al. [143], based on which these latter authors cautioned against targeting KA for therapeutic interventions to avoid adverse consequences, at least until further in-depth analysis of the interference of KA with various immune-related signaling pathways is made. It is noteworthy in the context of the AhR receptor that Kyn binding regulates the expression of the IL-10-RA (interleukin 10 receptor alpha subunit) in intestinal epithelia thus affording acceleration of IL-10-dependent wound closure [154]. Whether KA binding to the AhR can cause similar changes is currently unknown.

*Tryptophan 2,3-dioxygenase and indoleamine 2,3-dioxygenase* As stated in 3.2. above, TDO inhibition has been suggested as an antidepressant strategy, either alone or in combination with Trp. In addition to antidepressant drugs which also inhibit TDO activity, many other compounds used as adjuncts to or augmenters of antidepressant medication also inhibit TDO and some have been demonstrated to exert an antidepressant effect of their own or to accelerate clinical response to established antidepressants [34].

TDO inhibition may also play a role in combating anxiety and neurodegeneration in conditions such as Alzheimer’s Huntington’s and Parkinson’s diseases. Deletion of the TDO gene results in decreasing anxiety levels in mice, stimulation of neurogenesis and enhanced memory [68, 71]. As far as I could ascertain, no corresponding studies with TDO inhibitors in these human conditions have been performed.

TDO inhibition could also form the basis of therapy of acute hepatic porphyria. As stated in 6.3.3. above, liver TDO utilises the small haem pool that regulates haem biosynthesis by repression of 5-ALAS. Prevention of this utilisation enables the regulatory haem pool to exert its feedback control of its own synthesis. This prevention involves inhibition or prevention of the conjugation of apo-TDO with haem. There is evidence that treatments which cause this prevention are effective therapies of acute porphyric attacks. One such treatment is glucose, which is used in acute porphyric attacks precipitated by fasting. In rats, haem utilisation by TDO is enhanced by starvation via glucocorticoid induction of the apoenzyme [93]. Other precipitants of acute porphyric attacks are drugs, which are thought to act by direct 5-ALAS induction. Such induction may be overcome if availability of the regulatory haem pool is increased by inhibition of TDO conjugation with haem. TDO inhibition is therefore a potential new strategy for therapy of the hepatic porphyrias. Currently, therapy involves intravenous haem preparations or glucose.

TDO inhibition for cancer therapy has recently been explored at the experimental level. IDO1 inhibition for cancer therapy has been explored much earlier and will be discussed here along with TDO inhibition. Emphasis on TDO inhibition was prompted by 2 major observations: (1) IDO1 inhibition is not always effective in arresting the growth of tumors expressing IDO1; (2) demonstration of TDO expression in certain tumors. The association of IDO1 with cancer has arisen following the discovery of the immunomodulatory properties of this enzyme. Human cancerous tissue expression of IDO1, IDO2 and TDO2 has been studied [155]. Many types of cancers express IDO1, whereas that of IDO2 is negligible. TDO2 is also expressed in many cancers, but at lower levels than IDO1, except in hepatocellular carcinoma, where expression is considerably higher among all tissues examined and relative to that of IDO1. IDO1 inhibition has been an active targeting area for cancer therapy. Many IDO1 [156–158] and some TDO [159–161] inhibitors have been and continue to be developed. These and others have recently been reviewed [162]. Many issues need to be considered in developing IDO1 inhibitors [163], some of which are also applicable to TDO2 inhibitors. Among these issues is the inhibitory mechanism and its confirmation *in vivo*. Many IDO/TDO inhibitors act by a Trp competitive mechanism. Additionally, as stated above [77, 78], IDO is inhibited by [Trp] > 50 *μ*M. An excess of Trp could therefore undermine IDO1 inhibition in tumors. By contrast, the greater capacity of TDO for Trp will not cause reversal of the TDO inhibition by TDO inhibitors, unless too excessive Trp levels are reached in tumors. However, the increase in plasma [Trp] resulting from TDO inhibition in the host liver can lead to Trp accumulation in tumors. Given the discussion in 6.4.1. above and the detailed account in [13], a prudent strategy is to block Trp transport into tumors as a first line of attack and in conjunction with IDO1/TDO2 inhibition. Some negative or moderately successful outcomes of clinical trials with such inhibitors may be explained by tumoral immune escape based on the above issues. Also, as emphasized by Platten *et al* [160], preclinical studies have not considered exploring potential mechanisms of this immune escape. The proposed targeting of Trp availability to tumors [13] is one such approach in this direction. Furthermore, current trials do not select patients based on IDO1 expression in tumor tissue or assessment of systemic IDO1 activity by analysis of Trp and its metabolites in patients’ serum [161].

#### 6.4.3 N’-Formylkynurenine formamidase

A wide range of compounds inhibit activity of this enzyme, including organophosphate insecticides and metal cations [54]. Of the latter, Ag^+^ is the strongest inhibitor, causing 89% inhibition at 10 *μ*M [164]. However, most of these inhibitors are not suitable for use in humans and those that can be used in humans are weak ones. Although NFK formamidase activity is normally abundant in liver and possibly also other tissues, its inhibition can still block the formation of Kyn and its metabolites. Such inhibition could achieve the same aims as that of TDO and IDO and, in fact, development of formamidase inhibitors based on structure of the enzyme has been suggested [165].

#### 6.4.4 Kynurenine aminotransferase

As stated in 6.1. above, the KAT reactions (K → KA and 3-HK → XA) are minor ones limited by availability of the substrates because of the high K_*m*_s of both KAT A and KAT B respectively for their respective substrates. There are 4 KAT isoenzymes (KATs I, II, III and IV) with wide tissue distribution, of which KAT II has the highest activity in brain (60%). The importance of KAT in brain stems from the fact that it catalyses the formation of the NMDA receptor antagonist KA [117], which is cytoprotective and may thus be beneficial for cognitive function in patients with neurodegenerative and other CNS diseases. Elevation of KA levels in brain and CSF of patients with schizophrenia, however, induces a state of glutamatergic hypoactivity [166, 167]. Targeting KAT in schizophrenia has therefore been an active area of research [168–170] and detailed accounts of drug development of KAT inhibitors have been published [171–173]. As well as minimising glutamatergic hypoactivity, KAT inhibition also reduces the activity of mid-brain dopamine neurons [174], which is enhanced in schizophrenia.

KAT is inhibited by hydrazine compounds, some of which are used in medicine, e.g. the anti-tuberculous drug isonicotinic acid hydrazide, which causes a 92% inhibition when given in the rat’s diet at a 0.05% concentration [175]. The peripheral aromatic *L*-amino acid decarboxylase (ALAAD) inhibitors carbidopa and benserazide, which are used in Parkinson’s disease in conjunction with *L*-Dopa, also inhibit KAT activity both *in vitro* and after administration to rats [119]. When tested *in* vitro, carbidopa is twice as effective as benserazide (67% vs 33% inhibition at 25 *μ*M). KAT inhibition by chronic benserazide administration leads to accumulation of Kyn and 3-HK in liver and decreases in [KA] and [XA] in serum. These results suggest that benserazide may be useful in the treatment of schizophrenia. Two clinical trials have, however, produced negative results [176, 177], nevertheless, the decrease in [KA] [119] warrants further exploration of the potential clinical utility of benserazide in schizophrenia.

The above and other hydrazine compounds inhibit pyridoxal 5’-phosphate (PLP)-dependent enzymes by forming hydrazones with the cofactor, thus preventing the latter from binding to the enzymes [178]. Oestrogens and their derivatives can also inactivate PLP-dependent enzymes, e.g. KAT [179]. Thus, oestradiol disulphate is a strong inhibitor of KAT II (IC_50_ = 26.3 *μ*M), whereas oestradiol itself is a much weaker inhibitor (IC_50_ > 2 mM). It thus appears that the 17-sulphate moiety in oestradiol disulphate confers a much greater inhibitory potency. The authors [179] suggested that this can also be exploited in the design of novel KAT II inhibitors and can also contribute to improvement of existing inhibitors.

Inhibition of KAT B leading to decreased formation of XA may represent an important strategy for combating malaria. XA is the gametocyte-activating agent [129, 180] and while developmental efforts may be aimed at vaccine therapy, exploring the mechanism of gametocyte activation by XA may improve our understanding of the physiology of this parasite and lead to drug development including that aimed at blocking XA formation.

The 2 KAT isoenzymes responsible for KA formation in brain astrocytes are KAT I and KAT II and their targeting can be useful in treatment of schizophrenia. Inhibitors of these isoenzymes have been reviewed [162]. KAT I inhibitors are mainly Trp derivatives, whereas KAT II inhibitors include Kyn derivatives, fluoroquinolones and hydroxamate derivatives.

#### 6.4.5 Kynurenine monooxygenase (Kynurenine hydroxylase)

KMO has been the subject of intense research as a potential target for development of pharmacotherapy of many conditions, notably those affecting CNS function. A glance at Figure 4 will show that KMO inhibition should result in the following important changes in KP metabolite formation in liver, plasma, brain and/or CSF: (1) accumulation of Kyn; (2) consequent to the increase in this substrate, production of KA by the KAT A and of AA by the kynureninase A reactions is increased; (3) decreased formation of 3-HK; (4) a consequent decrease in production of subsequent metabolites from the main oxidative route, namely 3-HAA, QA and PA and from the transamination arm leading to XA formation by KAT B. These changes, which have important implications for drug development for a variety of disease states, can be seen from published data of the effects of the KMO inhibitors *m*-nitrobenzoylalanine in rats [181, 182] and mice [183] and 3,4-dimethoxy-N-[4-(3-nitrophenyl)thiazol-2-yl]benzenesulfonamide (Ro-61-8048) in mice [184], and of the effects of targeted deletion of the KMO gene in mice [185, 186]. These changes are desirable for achieving protection against the patho-physiological disturbances encountered in many disease states. A variety of studies have been reported on KMO inhibition in disease models. These include: (1) prolonged survival of mice with cerebral malaria by KMO inhibition by Ro-61-8048 [184]; (2) reduction of neuropathic pain in rats by the KMO inhibitors Ro-61-8048 and the pro-drug JM6 [2-(3,4-dimethoxybenzenesulfonylamino)-4-(3- nitrophenyl)-5-(piperidin-1-yl) methylthiazole,^187^]; (3) prevention by JM6 of spacial memory deficits, anxiety behaviour and synaptic loss in a mouse model of Alzheimer’s disease and extension of life span, prevention of synaptic loss and reduction of microglial activation in a mouse model of Huntington’s disease [188]; (4) prevention of multi-organ failure in a rat model of acute pancreatitis by the potent and specific KMO inhibitor the oxazolidine compound GSK 180 [189]. The status of current and new KMO inhibitors has been reviewed [162, 190] and it remains to be seen if or when some of these will be shown to be therapeutically effective in neurodegenerative diseases.

The accumulation of Kyn following KMO inhibition will increase both KA and AA levels. While increasing [KA] may not be desirable in schizophrenia, it can provide neuronal protection by combating the excitotoxicity of QA and reducing inflammation in inflammatory disease. The AA elevation may be of interest in relation to inflammatory disease, schizophrenia and diabetes and, as discussed earlier, as well as acting via its powerful apoptotic metabolite 5-HAA, AA can also decrease QA formation by inhibiting 3-HAAO [191].

The potential antiinflammatory consequences of KMO inhibition extend further down the KP via decreased production of 3-HK, 3-HAA and QA, given their pro-inflammatory properties discussed above in 6.3.4. Decreased formation of these Kyn metabolites by KMO inhibition can also prevent tumoral immune escape and it is noteworthy that KMO is upregulated in hepatocellular carcinoma [192] and triple negative breast cancer [193]. The potential impact of KMO inhibition on XA formation remains to be explored.

#### 6.4.6 Kynureninase

Kynureninase inhibition will increase the concentrations of Kyn, KA, 3-HK and XA, and decrease those of AA, 3-HAA and QA. Some of these effects have been reported [181, 182]. The kynureninase inhibitor nicotinylalanine has been reported [194] to inhibit the increase in rat brain [QA] induced by endotoxin administration. Among current therapeutic agents are the peripheral ALAAD inhibitors benserazide and carbidopa used in Parkinson’s disease (see 3.2. above). Both drugs are also kynureninase inhibitors, but, because they also inhibit KAT activity (see 6.4.5. above), the expected elevations of KA and XA do not occur [119]. Potent inhibitors of kynureninase have been synthesized [195] and patented [196] but await further investigation.

#### 6.4.7 3-Hydroxyanthranilic acid 3,4-dioxygenase

Inhibition of 3HAAO will block the formation of the excitotoxic metabolite QA and the antiinflammatory metabolite PA and cause accumulation of 3-HAA. While lowering [QA] may be desirable, increasing [3-HAA] is not, as it possesses proinflammatory properties. 3-HAAO inhibition may be justified if the enzyme is overexpressed, as has been demonstrated in the hepatic proteome of the hyperlipidaemic substance for normal cell function and integrity mouse HcB19 model [197] and brain of the triple transgenic Alzheimer’s Disease mouse model [198]. Even here, a better strategy is to limit 3-HAA availability by blocking the KP at an earlier step, namely that involving KMO.

#### 6.4.8 ACMSD (Picolinate carboxylase)

As described in 6.1. above, ACMSD converts ACMS to AMS, which can either undergo non-enzymic cyclisation to PA or continued degradation to acetyl CoA. Inhibition of ACMSD will therefore divert ACMS towards QA formation and subsequent NAD^+^ synthesis. NAD^+^ is a vital substance for maintenance of normal cell activity and functions, including cell signaling and gene regulation, and a decrease in its levels is associated with a variety of disease states. Pharmacological enhancement of NAD^+^ synthesis or availability is therefore an important goal. Recently, a potent ACMSD inhibitor, TES 1025, with an IC_50_ of 13 nM, has been developed and was shown to increase cellular NAD^+^ levels [199]. The application of this inhibitor to disease therapy remains to be seen.

Phthalate esters, especially di(2-ethylhexyl) phthalate and its metabolite mono (2-ethylhexyl) phthalate, MEHP), have been shown [200] to increase tissue levels of QA by inhibiting ACMSD. The authors suggested that phthalate ester toxicity could involve excessive QA production if a Trp-rich diet is consumed simultaneously. While increasing NAD^+^ levels may be desirable in some situations, a sustained inhibition of ACMSD activity may be undesirable, as it can lead to accumulation of QA with a resulting potential excitotoxicity. A genetic mutation of ACMSD has been observed in a family with Parkinson’s disease [201]. This latter group have also shown [202] that deletion of the ACMSD gene leads to a 20-50-fold increase in [QA] in liver, kidney, brain and plasma and suggested that this could be a suitable model for studies that can lead to new therapies of depression and neurodegenerative diseases.

#### 6.4.9 Enzymes of the pathways for NAD^+^ synthesis and consumption

The balance between activity of the NAD^+^-biosynthetic (the *de novo* and salvage) pathways and that of pathways of its consumption determines tissue NAD^+^ levels. As stated in 6.3.7. above, an adequate supply of NAD^+^ is vital for cell function at many levels, with shortages being associated with negative health consequences. Thus, NAD^+^ controls cellular metabolism and energy production. Processes such as glycolysis, the citric acid cycle, fatty acid and amino acid oxidation, the pentose phosphate pathway, biosynthetic processes and biological oxidation via cytochrome *P*-450 are all controlled by the NAD^+^(P^+^) H couples. The review by Srivastava [203] is informative. Lack of NAD^+^ can result in oxidative damage, DNA damage, muscle degeneration, inflammatory responses and age-related diseases [204, 205]. A variety of studies have implicated enzymes of NAD^+^ synthesis and utilisation in disease states. Examples include QPRT suppression of spontaneous cell death by lowering the overproduction of active caspase-3 [206], potential targeting of *Mycobacterium tuberculosis* with high affinity inhibitors of NAD synthetase, which is essential for survival of this pathogen [207], development of activators of NMNAT/NAMNAT for neuronal protection [208], over expression of this latter enzyme protects against acute CNS neurodegeneration by inhibiting excitotoxic-necrotic cell death [209], potential use of inhibitors of NMPRT in antitumor therapy by blocking glycolysis at the glyceraladehyde-3-phosphate dehydrogenase step [210]; the potential use of micro-RNA 26b as a suppressor of colorectal cancer tumor by inhibiting NMPRT [211], and use of NNMT as a target for treatment of obesity and type II diabetes [212].

NADase (NAD glycohydrolase) and streptolysin O are 2 proteins secreted by group A streptococci which promote virulence [213] and could therefore be potential targets for suppressing virulence [214]. Carbocyclic NAD analogues have been proposed as NADase inhibitors [215].

PARPs [poly(ADP-ribose) polymerases] is a family of enzymes that catalyse the transfer of ADP-ribose to target proteins, thus influencing many important processes, e.g. chromatin structure, transcription, replication, recombination, and DNA repair [216]. The latter effect is of special interest, as tumors may rely on this property of PARPs for survival. PARP inhibitors are therefore potential cancer therapeutic agents. Preclinical studies have shown PARP inhibitors in combination with other drugs or ionising radiation are effective against tumors and clinical trials are already in progress [217]. Preclinical data also suggest that PARP inhibitors may be effective therapies for non-cancer conditions, including stroke, neurotrauma, circulatory shock and acute myocardial infarction [217]. Other NAD^+^-consuming enzymes are sirtuins and c-ADP ribose synthases, both of which play major roles in control of NAD^+^ homeostasis, thus impacting many vital functions and are amenable to pharmacological interventions [203].

## 7 Conclusions and General Comments

The above account has demonstrated the multiple roles the essential amino acid *L*-tryptophan and its various metabolites play in health and disease and the wide range of scientific disciplines and medical specialties for which Trp metabolism is of special importance. Trp research over the past 7 decades has provided and continues to provide important information expanding our knowledge of physiological processes and facilitating our understanding of many disease states, ranging from abnormal behaviour and mental illness to carcinogenesis. Its unique involvement in health and disease has rendered Trp metabolism a fertile area for pharmacotherapeutic interventions and pharmacologists and other biomedical researchers can play an important role in drug developmental efforts for addressing many disease states. It is hoped that this review will stimulate interest among researchers in further enriching our knowledge of Trp and utilising it in disease therapy.

## Figures and Tables

**Table 1:** Role of tryptophan and its metabolites in various disciplines.

Discipline	Area	Metabolite, role and example
Basic sciences	Mammalian biochemistry	Trp and metabolites and various body systems
	Insect biochemistry	3-HK → xanthommatin in Drosophila
	Plant biochemistry	IAA as plant hormone (auxin)
	Behavioural science	IPA, 5-HT, KA, QA
	Immunology	Kyn and its metabolites (KA, QA, 3-HK, 3-HAA) as immunomodulators
	Neurochemistry	Trp, 5-HT, KA, QA
	Nutrition	Trp, NA, NAM, QA
	Pharmacology	5-HT receptor modulators
	Physiology	KA and QA as NMDA receptor modulators; melatonin and circadian rhythm
Medical Specialties	Cardiology	KA and inflammatory response
	Diabetes	XA, PA, AA, QA
	Gastroenterology	Serotonin, kynurenine metabolites in irritable bowel syndrome
	Hepatology	Trp in hepatic cirrhosis and encephalopathy
	Obs & Gynaecology	Trp utilization, Kyn metabolites as immunosuppressants
	Oncology	Immunosuppressive kynurenine metabolites
	Ophthalmology	Kyn and 5-HAA elevations and 3-HK photo-oxidation in cataract
	Parasitology	XA in malaria and IPA in trypanosomiasis
	Rheumatology	Kyn metabolites elevation after IDO induction
	Urology	Kyn metabolite elevation
	Veterinary medicine	Trp metabolism in herbivores
	Virology and other infections	Kyn metabolite elevation by IDO induction
Psychiatry	Alcoholism	5-HT deficiency, KA, 3-HK and 3-HAA as aversive agents
	Anxiety	5-HT, KA, QA
	Depression	5-HT
	Drug dependence	5-HT, KA
	OCD	5-HT
	Schizophrenia	KA
Neurological disease	Alzheimer’s disease	Kyn metabolites as immunomodulators
	Chronic brain injury	Kyn metabolites as immunomodulators
	Huntington’s disease	Kyn metabolites as immunomodulators
	Stroke	Kyn metabolites as immunomodulators

Moﬆ of the relevant sources are referenced in the liﬆ of references. Other sources can be accessed through search engines. Abbreviations used: AA (anthranilic acid), 3-HAA (3-hydroxyanthranilic acid), 5-HAA (5-hydroxyanthranilic acid), 3-HK (3-hydroxykynurenine), 5-HT (5-hydroxytryptamine or serotonin), IAA (indoleacetic acid), IDO (indoleamine 2,3-dioxygenase), IPA (indolepyruvic acid), KA (kynurenic acid), Kyn (kynurenine), NMDA (N-methyl-D-aspartate), NAM (nicotinamide), NA (nicotinic acid), PA (picolinic acid), QA (quinolinic acid).

**Table 2:** Plasma tryptophan disposition.

Parameter	Change	Mechanism	Examples of effectors
Free Trp	Decrease	TDO/IDO induction	Glucocorticoids/interferon-*γ*
		Inhibition of lipolysis	Insulin, nicotinic acid, antilipolytic agents
	Increase	TDO inhibition	glucose, nicotinamide, antidepressants
		Displacement from albumin	NEFA, catecholamines, ethanol, salicylate
		Decreased albumin	Pregnancy, liver and kidney diseases

Total Trp	Decrease	TDO/IDO induction	Glucocorticoids/interferon-*γ*
	Increase	TDO inhibition	glucose, nicotinamide, antidepressants

% Free Trp	Unaltered	TDO/IDO induction, TDO inhibition	
	Decrease	Increased albumin binding	
	Increase	Decreased albumin binding	

Reproduced here from Table 2 in ref [12] A. A.-B. Badawy. Tryptophan metabolism, disposition and utilisation in pregnancy. Biosci Rep. 35, art: be00261 / doi 10.1042/BSR20150197, 2015. Abbreviations used: IDO (indoleamine 2,3-dioxygenase), NEFA (non-esterified fatty acids), TDO (tryptophan 2,3-dioxygenase, formerly tryptophan pyrrolase)), Trp (tryptophan). The % free Trp is an expression of Trp binding to albumin and is = 100 X [free Trp]/[total Trp].

**Table 3:** Pharmacological targeting of the serotonin, tryptamine and indolepyruvate pathways.

Pathway	Enzyme/metabolite	Intended change	effector	Condition(s)
Serotonin	TPH2	activation	Trp	depression, anxiety
	TPH1	inhibition	various	osteoporosis, irritable bowel syndrome, carcinoid syndrome, obesity, ulcerative colitis, pulmonary arterial hypertension
	ALAAD	inhibition	carbidopa, benserazide	Parkinson’s disease
	MAO	inhibition	tranylcypromine and other MAOI	depression, Parkinson’s disease, panic and post-traumatic stress disorders, prostate cancer
	Melatonin	various uses	melatonin	autism spectrum disorders, cancer, mitochondrial protection, sleep
Tryptamine	Tryptamine	halogenation	derivatives to monitor brain function	Parkinson’s, Alzheimer’s schizophrenia
		Biotransformation	derivatives as hallucinogens	drug dependence
Indolepyruvate	Indolepyruvic acid	antioxidant	IPA, KA	epilepsy, cerebral ischaemia, Alzheimer’s disease
		Inhibition of IL-1β	IPA	overcoming immune escape by Trypanosomes

Abbreviations used: ALAAD (aromatic *L*-amino acid decarboxylase), IL-1β (interleukin-1β) IPA (indolepyruvic acid), KA (kynurenic acid), MAO (monoamine oxidase), MAOI (monoamine oxidase inhibitors), TPH1 and TPH2 (isoforms of tryptophan hydroxylase), Trp (tryptophan).

**Table 4:** Functions of the kynurenine pathway.

Function	Mediator (mechanism)
Detoxification of tryptophan	TDO (Glucocorticoid induction)
Control of plasma tryptophan availability	TDO (Trp flux and oxidation)
Control of liver haem biosynthesis	TDO (Utilisation of the regulatory haem pool)
Modulation of the immune system	Kyn, KA, 3-HK, 3-HAA, QA, PA (Cytokine induction of IDO)
Modulation of carbohydrate metabolism	XA, PA (Binding of insulin and Zn); QA (inhibition of PEPCK)
Pellagra prevention	QA, Nicotinic acid, nicotinamide (NAD^+^ synthesis)
NAD^+^ synthesis	QA, nicotinic acid, nicotinamide (*De novo* and Salvage pathways)

Abbreviations used: 3-HAA (3-hydroxyanthranilic acid), 3-HK (3-hydroxykynurenine), IDO (indoleamine 2,3-dioxygenase), KA (kynurenic acid), Kyn (kynurenine), NAD^+^ (oxidized nicotinamide-adenine dinucleotide), PEPCK (phosphoenolpyruvate carboxykinase), PA (picolinic acid), QA (quinolinic acid), TDO (tryptophan 2,3-dioxygenase), Trp (tryptophan), XA (xanthurenic acid).

**Table 5:** Pharmacological targeting of the kynurenine pathway.

Target	Condition	Mechanisms and effectors
Trp and metabolites:		
Trp	tumoral immune escape	Decrease tumoral uptake by α-MT
		Decrease plasma free Trp by albumin infusions
		Decreased plasma free Trp by antilipolytic agents
Nicotinic acid	cancer	Decreased plasma free Trp by inhibition of lipolysis in host
Nicotinamide	cancer	TDO inhibition in tumors and hosts
Kynurenic acid	Alcoholism	Aversion to alcohol by ALDH inhibition
	Schizophrenia	KAT inhibition
	Inflammatory diseases	KA analogues
	Retinal degeneration	Increasing KA formation by Trp?
	GIT diseases	Increasing KA formation by Trp
Enzymes:		
TDO	Depression	Increased serotonin synthesis by TDO inhibition (antidepressants, others)
	Neurodegeneration	Increased KA formation
	Anxiety	Increased brain serotonin by TDO inhibition
	Hepatic porphyrias	Decreased haem utilisation by TDO inhibitors (glucose, others?)
	Cancer, immune escape	Decreased immunosuppressive kynurenines by TDO inhibitors
IDO	cancer, immune escape	Decreased immunosuppressive kynurenines by IDO inhibitors
Formamidase	Neurological diseases	Decreasing neurotoxic Kyn metabolites by formamidase inhibition
	Cancer and infections	Decreasing immunosuppressive Kyn metabolites by formamidase inhibition
KAT	Schizophrenia	Improved glutamatergic activity by KAT II inhibition
	Malaria infection	Decreasing XA by KAT inhibition
KMO	anxiety, cerebral malaria, Inflammatory and neurodegenerative diseases, pancreatitis	Decreased 3-HK, 3-HAA and QA and increased Kyn and KA by KMO inhibitors
Kynureninase	Neurodegenerative diseases	Decreased 3-HAA and QA formation by kynureninase inhibition
3-HAAO	Neurodegenerative diseases	Decreased QA formation by 3-HAAO inhibition
ACMSD	No definite views	Inhibition could be controversial
QPRT	neurodegenerative Diseases	Stimulation to lower QA
	Cancer	Inhibition to decrease NAD^+^ to undermine tumor viability
		And suppression of cell death by inhibition of caspase production
NAD synthetase	Mycobacterium tuberculosis	Inhibition to limit NAD^+^ availability
NMPRT	Cancer	Inhibition to suppress colorectal tumors
NMNAT-NAMNAT	Neurological and neuro-degenerative diseases	Activation to increase NAD^+^ synthesis to combat oxidative damage
NNMT	Obesity, type 2 diabetes	Inhibition undermines processes related to glucose metabolism and fat deposition
NADase	Streptococcal virulence	Inhibition of NADase
PARP	Cancer, stroke, myocardial infarction, neurotrauma	Inhibition of PARP activity

Abbreviations used: ACMSD (2-amino-3-carboxymuconic acid-6-semialdehyde; also known as acroleyl aminofumarate), 3-HAA (3-hydroxyanthranilic acid), 3-HAAO (3-hydroxyanthranilic acid 3,4-dioxygenase), 3-HK (3-hydroxykynurenine), IDO (indoleamine 2,3-dioxygenase), KA (kynurenic acid), Kyn (kynurenine), KAT (kynurenine aminotransferase), KMO (kynurenine monooxygenase or kynurenine hydroxylase), α-MT (α-methyltryptophan), NAMNAT/NMNAT (nicotinamide mononucleotide/nicotinic acid mononucleotide adenylyl transferases), NMPRT (nicotinamide phosphoribosyltransferase), NNMT (nicotinamide N-methyltransferase), PARP [poly (ADP-ribose) polymerase], QPRT (quinolinate phosphoribosyltransferase), QA (quinolinic acid), Trp (tryptophan), TDO (tryptophan 2,3-dioxygenase), XA (xanthurenic acid).

## References

[1] Amer MS, Abdel-Daim MH, Abdel-Tawab GA (1968). Studies with tryptophan metabolites in vitro. Kynurenine
metabolism in kidneys of mice infested with Schistosoma
mansoni. Biochemical Journal.

[2] Kelada FS, Abdel-Tawab GA, Moustafa MH, Konbar AA (1972). Comparative studies on the in vivo effects of tartar emetic,
vitamin B6, and the chelating agent 2,3-dimercaptopropanol (BAL) on the
functional capacity of the tryptophan-niacin pathway in patients with
schistosomiasis. Metabolism.

[3] Saad AA, Abdel-Tawab GA, El-Zoghiby SM, Mostafa MH, Moursi GE (1974). Relationship between pyridoxal phosphate and some synthetic
oestrogens in their effect on kynurenine hydrolase and kynurenine
aminotransferase enzymes of normal mouse liver. Biochemical Pharmacology.

[4] El-Zoghby SM, El-Sewedy SM, Saad AA, Mostafa MH, Ebied SM, Abdel-Tawab GA (1976). In vitro Trials to counteract the inhibitory effect of
β-oestradiol and ethynyloestradiol on the B6-dependent kynurenine
aminotransferase enzyme. Biochemical Pharmacology.

[5] Hayaishi O (1993). My life with tryptophan—Never a dull
moment. Protein Science.

[6] Badawy AA-B (2017). Kynurenine Pathway of Tryptophan Metabolism: Regulatory and
Functional Aspects. International Journal of Tryptophan Research.

[7] Bender DA (1983). Biochemistry of tryptophan in health and disease. Molecular Aspects of Medicine.

[8] Badawy AA-B (2010). Plasma free tryptophan revisited: What you need to know and do
before measuring it. Journal of Psychopharmacology.

[9] Morgan CJ, Badawy AA-B (1994). Effects of storage on binding and stability of tryptophan in
human serum. Annals of Clinical Biochemistry.

[10] Denckla WD, Dewey HK (1967). The determination of tryptophan in plasma, liver and
urine. J Lab Clin Med.

[11] Bloxam DL, Warren WH (1974). Error in the determination of tryptophan by the method of Denckla
Dewey: a revised procedure. Anal Biochem.

[12] Badawy AA-B (2015). Tryptophan metabolism, disposition and utilisation in
pregnancy. Biosci Rep.

[13] Badawy AA (2018). Targeting tryptophan availability to tumors: the answer to immune
escape?. Immunology & Cell Biology.

[14] Carlsson A, Lindqvist M (1978). Dependence of 5-HT and catecholamine synthesis on concentrations
of precursor amino-acids in rat brain. Naunyn-Schmiedeberg’s Archives of Pharmacology.

[15] Friedman PA, Kappelman AH, Kaufman S (1972). Partial purification and characterization of tryptophan
hydroxylase from rabbit hindbrain. The Journal of Biological Chemistry.

[16] Grahame-Smith DG (1971). Studies in vivo on the relationship between brain tryptophan,
brain 5-ht synthesis and hyperactivity in rats treated with a monoamine
oxidase inhibitor and L-tryptophan. Journal of Neurochemistry.

[17] Kaufman S (1974). Properties of the Pterin-Dependent Aromatic Amino Acid
Hydroxylases.

[18] Badawy AA-B, Williams DL (1982). Enhancement of rat brain catecholamine synthesis by
administration of small doses of tyrosine and evidence for substrate
inhibition of tyrosine hydroxylase activity by large doses of the amino
acid. Biochemical Journal.

[19] Gál EM, Young RB, Sherman AD (1978). Tryptophan loading: consequent effects on the synthesis of
kynurenine and 5-hydroxyindoles in rat brain. Journal of Neurochemistry.

[20] Young SN, Smith SE, Pihl RO, Ervin FR (1985). Tryptophan depletion causes a rapid lowering of mood in normal
males. Psychopharmacology.

[21] Badawy AA-B, Dougherty DM, Richard DM (2010). Specificity of the acute tryptophan and tyrosine plus
phenylalanine depletion and loading tests part II: Normalisation of the
tryptophan and the tyrosine plus phenylalanine to competing amino acid
ratios in a new control formulation. International Journal of Tryptophan Research.

[22] Robins E, Robins JM, Croninger AB, Moses SG, Spencer SJ, Hudgens RW (1967). The low level of 5-hydroxytryptophan decarboxylase in human
brain. Biochemical Medicine.

[23] Hardeland R (2017). Melatonin — More than Just a Pineal
Hormone. Biomedical Journal of Scientific & Technical Research.

[24] Chowdhyry I, Sengupta A, Maitra SK (2008). Melatonin, 50 years of scientific journey from the discovery in
bovine pineal gland to delineation of functions in humans. Indian J Biochem Biophys.

[25] Tan D, Xu B, Zhou X, Reiter R (2018). Pineal Calcification, Melatonin Production, Aging, Associated
Health Consequences and Rejuvenation of the Pineal Gland. Molecules.

[26] Bernard M, Guerlotté J, Grève P (1999). Melatonin synthesis pathway: Circadian regulation of the genes
encoding the key enzymes in the chicken pineal gland and
retina. Reproduction Nutrition Development.

[27] Sánchez S, Sánchez CL, Paredes SD, Rodriguez AB, Barriga C (2008). The effect of tryptophan administration on the circadian rhythms
of melatonin in plasma and the pineal gland of rats. Journal of Applied Biomedicine.

[28] Flamand V, Zhao H, Peehl DM (2010). Targeting monoamine oxidase A in advanced prostate
cancer. Journal of Cancer Research and Clinical Oncology.

[29] Wu JB, Shao C, Li X (2014). Monoamine oxidase A mediates prostate tumorigenesis and cancer
metastasis. The Journal of Clinical Investigation.

[30] Pisani L, Catto M, Leonetti F (2011). Targeting monoamine oxidases with multipotent ligands: An
emerging strategy in the search of new drugs against neurodegenerative
diseases. Current Medicinal Chemistry.

[31] Singh Y, Swanson E, Sokoloski E, Krishnan Kutty R, Krishna G (1988). MPTP and MPTP analogs induced cell death in cultured rat
hepatocytes involving the formation of pyridinium
metabolites. Toxicology and Applied Pharmacology.

[32] McCrodden JM, Tipton KF, Sullivan JP (1990). Minireview, The neurotoxicity of MPTP and the relevance to
Parkinsons disease. Pharmacol Toxicol.

[33] Torrente MP, Gelenberg AJ, Vrana KE (2012). Boosting serotonin in the brain: Is it time to revamp the
treatment of depression?. Journal of Psychopharmacology.

[34] Badawy AA-B (2013). Tryptophan: The key to boosting brain serotonin synthesis in
depressive Illness. Journal of Psychopharmacology.

[35] Badawy AA-B, Evans M (1974). Tryptophan plus a pyrrolase inhibitor for
depression?. The Lancet.

[36] Badawy AA-B, Evans M (1975). Tryptophan plus a pyrrolase inhibitor for
depression. The Lancet.

[37] Meltzer HY, Lowy MT, Meltzer HY (1987). The serotonin hypothesis of depression. Psychopharmacology: The Third Generation of Progress.

[38] Waløen K, Kleppe R, Martinez A, Haavik J (2017). Tyrosine and tryptophan hydroxylases as therapeutic targets in
human disease. Expert Opinion on Therapeutic Targets.

[39] Pagan C, Goubran-Botros H, Delorme R (2017). Disruption of melatonin synthesis is associated with impaired
14-3-3 and miR-451 levels in patients with autism spectrum
disorders. Scientific Reports.

[40] Talib W (2018). Melatonin and Cancer Hallmarks. Molecules.

[41] Tan D, Manchester L, Qin L, Reiter R (2016). Melatonin: A Mitochondrial Targeting Molecule Involving
Mitochondrial Protection and Dynamics. International Journal of Molecular Sciences.

[42] Khalil EM, De Angelis J, Ishii M, Cole PA (1999). Mechanism-based inhibition of the melatonin rhythm enzyme:
Pharmacologic exploitation of active site functional
plasticity. Proceedings of the National Acadamy of Sciences of the United States of
America.

[43] Jones RSG (1982). Tryptamine: a neuromodulator or neurotransmitter in mammalian
brain?. Progress in Neurobiology.

[44] Mousseau DD (1993). Tryptamine: A metabolite of tryptophan implicated in various
neuropsychiatric disorders. Metabolic Brain Disease.

[45] Young SN, Lal S (1980). CNS tryptamine metabolism in hepatic coma. Journal of Neural Transmission.

[46] Weissbach H, King W, Sjoerdsma A, Udenfriend S (1959). Formation of indole-3-acetic acid and tryptamine in animals: a
method for estimation of indole-3-acetic acid in tissues. The Journal of Biological Chemistry.

[47] Young SN, St-Arnaud-McKenzie D, Sourkes TL (1978). Importance of tryptophan pyrrolase and aromatic amino acid
decarboxylase in the catabolism of tryptophan. Biochemical Pharmacology.

[48] Young SN, Anderson GM, Gauthier S, Purdy WC (1980). The Origin of Indoleacetic Acid and Indolepropionic Acid in Rat
and Human Cerebrospinal Fluid. Journal of Neurochemistry.

[49] Williams BB, Van Benschoten AH, Cimermancic P (2014). Discovery and characterization of gut microbiota decarboxylases
that can produce the neurotransmitter tryptamine. Cell Host & Microbe.

[50] Pacak K, Eisenhofer G, Carrasquillo JA, Chen CC, Li S-T, Goldstein DS (2001). 6-[^18^F]fluorodopamine positron emission tomographic
(PET) scanning for diagnostic localization of
pheochromocytoma. Hypertension.

[51] Dragulska S, Kańska M (2014). Enzymatic synthesis of tryptamine and its halogen derivatives
selectively labeled with hydrogen isotopes. Journal of Radioanalytical and Nuclear Chemistry.

[52] Thierry FY, Monisola II, Berka NP, Derek NT (2017). Biological applications of tryptamine Schiff base
derivatives.

[53] Stanley JC, Nicholas AR, Dickson AJ, Thompson IM, Pogson CI (1984). Tryptophan aminotransferase activity in rat liver. Biochemical Journal.

[54] (2002). BRENDA: the comprehensive enzyme information
system. Choice Reviews Online.

[55] Ramos-Chávez LA, Lugo Huitrón R, González Esquivel D (2018). Relevance of Alternative Routes of Kynurenic Acid Production in
the Brain. Oxidative Medicine and Cellular Longevity.

[56] Berg CP, Rose WC, Marvel CS (1929). Tryptophane and growth. III. 3-indolepropionic acid and
3-indolepyruvic acid as supplementing agents in diets deficient in
tryptophane. The Journal of Biological Chemistry.

[57] O’Neil SR, DeMoss RD (1968). Tryptophan transaminase from Clostridium
sporogenes. Archives of Biochemistry and Biophysics.

[58] Richards P, Brown CL, Lowe SM (1972). Synthesis of tryptophan from 3-indolepyruvic acid by a healthy
woman. Journal of Nutrition.

[59] Bacciottini L, Pellegrini-Giampietro DE, Bongianni F, de Luca G, Politi V, Moroni F (1987). Biochemical and behavioural studies on indole-pyruvic acid: a
keto-analogue of tryptophan. Pharmacological Research Communications.

[60] Politi V, D’Alessio S, Di Stazio G, De Luca G (1996). Antioxidant Properties of Indole-3-Pyruvic Acid. Recent Advances in Tryptophan Research, vol. 398 of Advances in
Experimental Medicine and Biology.

[61] Patent 3-Indolepyruvic acid derivatives and pharmaceutical use
thereof. Proceedings of the PCT/IT88/00041. International publication number: WO
88/09789 15.12.88 Gazette 88/27.

[62] McGettrick AF, Corcoran SE, Barry PJ (2016). Trypanosoma brucei metabolite indolepyruvate decreases
HIF-1α and glycolysis in macrophages as a mechanism of innate immune
evasion. Proceedings of the National Acadamy of Sciences of the United States of
America.

[63] Williams JN, Feigelson P, Elvehjem CA (1950). Relation of tryptophan and niacin to pyridine nucleotides of
tissue. The Journal of Biological Chemistry.

[64] Bender DA, Magboul BI, Wynick D (1982). Probable mechanisms of regulation of the utilization of dietary
tryptophan, nicotinamide and nicotinic acid as precursors of nicotinamide
nucleotides in the rat. British Journal of Nutrition.

[65] Mccreanor GM (1986). The metabolism of high intakes of tryptophan, nicotinamide and
nicotinic acid in the rat. British Journal of Nutrition.

[66] Horwitt MK, Harvey CC, Rothwell WS, Cutler JL, Haffron D (1956). Tryptophan-Niacin Relationships in Man. Journal of Nutrition.

[67] Goldsmith GA (1958). Niacin-tryptophan relationships in man and niacin
requirement. American Journal of Clinical Nutrition.

[68] Kanai M, Funakoshi H, Takahashi H (2009). Tryptophan 2,3-dioxygenase is a key modulator of physiological
neurogenesis and anxiety-related behavior in mice. Molecular Brain.

[69] Terakata M, Fukuwatari T, Kadota E (2013). The niacin required for optimum growth can be synthesized from
L-tryptophan in growing mice lacking
tryptophan-2,3-dioxygenase. Journal of Nutrition.

[70] Santillan MK, Pelham CJ, Ketsawatsomkron P (2015). Pregnant mice lacking indoleamine 2,3-dioxygenase exhibit
preeclampsia phenotypes. Physiological Reports.

[71] Too LK, Li KM, Suarna C (2016). Deletion of TDO2, IDO-1 and IDO-2 differentially affects mouse
behavior and cognitive function. Behavioural Brain Research.

[72] Cho-Chung YS, Pitot HC (1967). Feedback control of rat liver tryptophan pyrrolase. I. End
product inhibition of trytophan pyrrolase activity. The Journal of Biological Chemistry.

[73] Badawy AA-B, Evans M (1976). The regulation of rat liver tryptophan pyrrolase activity by
reduced nicotinamide adenine dinucleotide (phosphate). Experiments with
glucose and nicotinamide. Biochemical Journal.

[74] Badawy AA-B (2002). Tryptophan metabolism in alcoholism. Nutrition Research Reviews.

[75] Yoshida R, Nukiwa T, Watanabe Y, Fujiwara M, Hirata F, Hayaishi O (1980). Regulation of indoleamine 2,3-dioxygenase activity in the small
intestine and the epididymis of mice. Archives of Biochemistry and Biophysics.

[76] Cook JS, Pogson CI, Smith SA (1980). Indoleamine 2,3-dioxygenase. A new, rapid, sensitive radiometric
assay and its application to the study of the enzyme in rat
tissues. Biochemical Journal.

[77] Ozaki Y, Reinhard JF, Nichol CA (1986). Cofactor activity of dihydroflavin mononucleotide and
tetrahydrobiopterin for murine epididymal indoleamine
2,3-dioxygenase. Biochemical and Biophysical Research Communications.

[78] Efimov I, Basran J, Sun X (2012). The mechanism of substrate inhibition in human indoleamine
2,3-dioxygenase. Journal of the American Chemical Society.

[79] Pfefferkorn ER, Rebhun S, Eckel M (1986). Characterization of an Indoleamine 2,3-Dioxygenase Induced by
Gamma-Interferon in Cultured Human Fibroblasts. Journal of Interferon Research.

[80] Ozaki Y, Edelstein MP, Duch DS (1987). The actions of interferon and antiinflammatory agents on
induction of indoleamine 2,3-dioxygenase in human peripheral blood
monocytes. Biochemical and Biophysical Research Communications.

[81] Hucke C, MacKenzie CR, Adjogble KDZ, Takikawa O, Däubener W (2004). Nitric Oxide-Mediated Regulation of Gamma Interferon-Induced
Bacteriostasis: Inhibition and Degradation of Human Indoleamine
2,3-Dioxygenase. Infection and Immunity.

[82] Thomas SR, Terentis AC, Cai H (2007). Post-translational regulation of human indoleamine 2,
3-dioxygenase activity by nitric oxide. The Journal of Biological Chemistry.

[83] Badawy AA-B, Evans M (1976). Animal liver tryptophan pyrrolases. Absence of apoenzyme and of
hormonal induction mechanism from species sensitive to tryptophan
toxicity. Biochemical Journal.

[84] Ikeda M, Tsuji H, Nakamura S, Ichiyama A, Nishizuka Y, Hayaishi O (1965). Studies on the biosynthesis of nicotinamide adenine dinucleotide.
II. A role of picolinic carboxylase in the biosynthesis of nicotinamide
adenine dinucleotide from tryptophan in mammals. The Journal of Biological Chemistry.

[85] Allegri G, Bertazzo A, Biasiolo M, Costa CVL, Ragazzi E (2003). Kynurenine pathway enzymes in different species of
animals. Advances in Experimental Medicine and Biology.

[86] Murakami Y, Saito K (2013). Species and cell types difference in tryptophan
metabolism. International Journal of Tryptophan Research.

[87] Horita A, Carino MA (1970). Modification of the toxic actions of L-tryptophan by pargyline
and p-chIorophenylalanine. Biochemical Pharmacology.

[88] Knox WE (1966). The regulation of tryptophan pyrrolase activity by
tryptophan. Advances in Enzyme Regulation.

[89] Badawy AA-B (2013). Tryptophan and inhibitors of tryptophan 2,3-dioxygenase as
antidepressants: Reply. Journal of Psychopharmacology.

[90] Muñoz-Clares RA, Lloyd P, Lomax MA, Smith SA, Pogson CI (1981). Tryptophan metabolism and its interaction with gluconeogenesis in
mammals: Studies with the guinea pig, mongolian gerbil, and
sheep. Archives of Biochemistry and Biophysics.

[91] Badawy AA-B, Evans M (1983). Opposite effects of chronic administration and subsequent
withdrawal of drugs of dependence on the metabolism and disposition of
endogenous and exogenous tryptophan in the rat. Alcohol and Alcoholism.

[92] Werner ER, Fuchs D, Hausen A (1988). Tryptophan Degradation in Patients Infected by Human
Immunodeficiency Virus. Biological Chemistry Hoppe-Seyler.

[93] Badawy AA-B (1979). Central role of tryptophan pyrrolase in haem
metabolism. Biochemical Society Transactions.

[94] Badawy AA-B, Morgan CJ (1980). Tryptophan pyrrolase in haem regulation. The relationship between
the depletion of rat liver tryptophan pyrrolase haem and the enhancement of
5-aminolaevulinate synthase activity by
2-allyl-2-isopropylacetamide. Biochemical Journal.

[95] Morgan CJ, Badawy AA-B (1980). Tryptophan pyrrolase in haem regulation. The mechanism of the
permissive effect of cortisol on the enhancement of 5-aminolaevulinate
synthase activity by 2-allyl-2-isopropylacetamide in the
adrenalectomized-rat liver. Journal of Pharmacy and Pharmacology.

[96] Welch AN, Badawy AA-B (1980). Tryptophan pyrrolase in haem regulation. Experiments with
administered haematin and the relationship between the haem saturation of
tryptophan pyrrolase and the activity of 5-aminolaevulinate synthase in rat
liver. Biochemical Journal.

[97] Badawy AA-B, Morgan CJ (1982). Tryptophan and tryptophan pyrrolase in haem regulation. The role
of lipolysis and direct displacement of serum-protein-bound tryptophan in
the opposite effects of administration of endotoxin, morphine, palmitate,
salicylate and theophylline on rat liver 5-aminolaevulinate synthase
activity and the haem saturation of tryptophan pyrrolase. Biochemical Journal.

[98] Badawy AA-B (1981). Heme utilization by rat liver tryptophan pyrrolase as a screening
test for exacerbation of hepatic porphyrias by drugs. Journal of Pharmacological Methods.

[99] Yamamoto S, Hayaishi O (1967). Tryptophan pyrrolase of rabbit intestine. D- and
L-tryptophan-cleaving enzyme or enzymes. The Journal of Biological Chemistry.

[100] Takikawa O, Yoshida R, Kido R, Hayaishi O (1986). Tryptophan degradation in mice initiated by indoleamine
2,3-dioxygenase. The Journal of Biological Chemistry.

[101] Yasui H, Takai K, Yoshida R, Hayaishi O (1986). Interferon enhances tryptophan metabolism by inducing pulmonary
indoleamine 2,3-dioxygenase: Its possible occurrence in cancer
patients. Proceedings of the National Acadamy of Sciences of the United States of
America.

[102] Pfefferkorn ER, Rebhun S, Eckel M (1986). Characterization of an Indoleamine 2,3-Dioxygenase Induced by
Gamma-Interferon in Cultured Human Fibroblasts. Journal of Inter-feron Research.

[103] Taylor MW, Feng G (1991). Relationship between interferon-γ, indoleamine
2,3-dioxygenase, and tryptophan catabolism. The FASEB Journal.

[104] Munn DH, Zhou M, Attwood JT (1998). Prevention of allogeneic fetal rejection by tryptophan
catabolism. Science.

[105] Badawy AA-B (1988). Effects of pregnancy on tryptophan metabolism and disposition in
the rat. Biochemical Journal.

[106] Moffett JR, Namboodiri MA (2003). Tryptophan and the immune response. Immunology & Cell Biology.

[107] Badawy AA-B (2014). The tryptophan utilization concept in pregnancy. Obstetrics & Gynecology Science.

[108] Badawy AA-B (2015). Tryptophan metabolism, disposition and utilization in
pregnancy. Bioscience Reports.

[109] Badawy AA-B, Namboodiri AMA, Moffett JR (2016). The end of the road for the tryptophan depletion concept in
pregnancy and infection. Clinical Science.

[110] Heyliger SO, Mazzio EA, Soliman KFA (1999). The anti-inflammatory effects of quinolinic acid in the
rat. Life Sciences.

[111] Terness P, Bauer TM, Röse L (2002). Inhibition of allogeneic T cell proliferation by indoleamine
2,3-dioxygenase–expressing dendritic cells: mediation of suppression
by tryptophan metabolites. The Journal of Experimental Medicine.

[112] Fallarino F, Grohmann U, Vacca C (2002). T cell apoptosis by tryptophan catabolism. Cell Death & Differentiation.

[113] Badawy AA-B (2018). Hypothesis kynurenic and quinolinic acids: The main players of
the kynurenine pathway and opponents in inflammatory disease. Medical Hypotheses.

[114] Moffett JR, Espey MG, Namboodiri MAA (1994). Antibodies to quinolinic acid and the determination of its
cellular distribution within the rat immune system. Cell & Tissue Research.

[115] Nishizuka Y, Hayaishi O (1963). Studies on the biosynthesis of nicotinamide adenine nucleotides
I. enzymatic synthesis of niacin ribonucleotides from 3-hydroxyanthranilic
acid in mammalian tissue. The Journal of Biological Chemistry.

[116] Bender DA (1980). Inhibition in vitro of the enzymes of the oxidative pathway of
tryptophan metabolism and of nicotinamide nucleotide synthesis by
benserazide, carbidopa and isoniazid. Biochemical Pharmacology.

[117] Stone TW (1993). The neuropharmacology of quinolinic and kynurenic
acids. Pharmacological Reviews.

[118] Darlington LG, Forrest CM, Mackay GM (2010). On the biological importance of the 3-hydroxyanthranilic acid:
anthranilic acid ratio. International Journal of Tryptophan Research.

[119] Badawy AA-B, Bano S (2016). Tryptophan Metabolism in Rat Liver after Administration of
Tryptophan, Kynurenine Metabolites, and Kynureninase
Inhibitors. International Journal of Tryptophan Research.

[120] Smith SA, Carr FP, Pogson CI (1980). The metabolism of L-tryptophan by isolated rat liver cells.
Quantification of the relative importance of, and the effect of nutritional
status on, the individual pathways of tryptophan metabolism. Biochemical Journal.

[121] Smith SA, Pogson CI (1977). Tryptophan and the control of plasma glucose concentrations in
the rat. Biochemical Journal.

[122] Reginaldo C, Jacques P, Scott T, Oxenkrug G, Selhub J, Paul L (2015). Xanthurenic acid is associated with higher insulin resistance and
higher odds of diabetes. FASEB J.

[123] Ikeda S, Kotake Y (1986). Urinary excretion of xanthurenic acid and zinc in diabetes: (3)
Occurrence of xanthurenic acid-Zn2+ complex in urine of diabetic patients
and of experimentally-diabetic rats. Italian Journal of Biochemistry.

[124] Oxenkrug GF (2015). Increased plasma levels of xanthurenic and kynurenic acids in
type 2 diabetes. Molecular Neurobiology.

[125] Chausmer AB (1998). Zinc, insulin, and diabetes. Journal of the American College of Nutrition.

[126] Zuwała-Jagiello J, Pazgan-Simon M, Simon K, Warwas M (2012). Picolinic Acid in Patients with Chronic Hepatitis C Infection: A
Preliminary Report. Mediators of Inflammation.

[127] Medana IM, Day NPJ, Salahifar-Sabet H (2003). Metabolites of the kynurenine pathway of tryptophan metabolism in
the cerebrospinal fluid of Malawian children with malaria. The Journal of Infectious Diseases.

[128] Grant R, Coggan S, Smythe G (2009). The Physiological Action of Picolinic Acid in the Human
Brain. International Journal of Tryptophan Research.

[129] Billker O, Lindo V, Panico M (1998). Identification of xanthurenic acid as the putative inducer of
malaria development in the mosquito. Nature.

[130] Gobaille S, Kemmel V, Brumaru D, Dugave C, Aunis D, Maitre M (2008). Xanthurenic acid distribution, transport, accumulation and
release in the rat brain. Journal of Neurochemistry.

[131] Badawy AA-B (2014). Pellagra and alcoholism: A biochemical
perspective. Alcohol and Alcoholism.

[132] Badawy AA-B, Lake SL, Dougherty DM (2014). Mechanisms of the Pellagragenic Effect of Leucine: Stimulation of
Hepatic Tryptophan Oxidation by Administration of Branched-Chain Amino Acids
to Healthy Human Volunteers and the Role of Plasma Free Tryptophan and Total
Kynurenines. International Journal of Tryptophan Research.

[133] Fukuwatari T, Shibata K (2013). Nutritional aspect of tryptophan metabolism. International Journal of Tryptophan Research.

[134] Jacob RA, Swendseid ME, McKee RW, Fu CS, Clemens RA (1989). Biochemical markers for assessment of niacin status in young men:
Urinary and blood levels of niacin metabolites. Journal of Nutrition.

[135] Shibata K, Matsuo H (1989). Correlation between niacin equivalent intake and urinary
excretion of its metabolites, N’-methylnicotinamide,
N’-methyl-2-pyridone-5-carboxamide, and
N’-methyl-4-pyridone-3-carboxamide, in humans consuming a
self-selected food. American Journal of Clinical Nutrition.

[136] Bhutia YD, Babu E, Ramachandran S, Ganapathy V (2015). Amino acid transporters in cancer and their relevance to
”glutamine addiction”: Novel Targets for the design of a new
class of anticancer drugs. Cancer Research.

[137] Bhutia YD, Babu E, Prasad PD, Ganapathy V (2014). The amino acid transporter SLC6A14 in cancer and its potential
use in chemotherapy. Asian Journal of Pharmaceutical Sciences.

[138] Karunakaran S, Umapathy NS, Thangaraju M (2008). Interaction of tryptophan derivatives with SLC6A14
(ATB^0,+^) reveals the potential of the transporter as a drug
target for cancer chemotherapy. Biochemical Journal.

[139] Karunakaran S, Ramachandran S, Coothankandaswamy V (2011). SLC6A14 (ATB^0,+^) protein, a highly concentrative and
broad specific amino acid transporter, is a novel and effective drug target
for treatment of estrogen receptor-positive breast cancer. The Journal of Biological Chemistry.

[140] Timosenko E, Ghadbane H, Silk JD (2016). Nutritional stress induced by tryptophan-degrading enzymes
results in ATF4-dependent reprogramming of the amino acid transporter
profile in tumor cells. Cancer Research.

[141] Kudo Y, Boyd CAR, Sargent IL, Redman CWG (2001). Tryptophan degradation by human placental indoleamine
2,3-dioxygenase regulates lymphocyte proliferation. The Journal of Physiology.

[142] Wang J, Simonavicius N, Wu X (2006). Kynurenic acid as a ligand for orphan G protein-coupled receptor
GPR35. The Journal of Biological Chemistry.

[143] Wirthgen E, Hoeflich A, Rebl A, Günther J (2018). Kynurenic Acid: The Janus-Faced Role of an Immunomodulatory
Tryptophan Metabolite and Its Link to Pathological
Conditions. Frontiers in Immunology.

[144] Tiszlavicz Z, Németh B, Fülöp F (2011). Different inhibitory effects of kynurenic acid and a novel
kynurenic acid analogue on tumour necrosis factor-α (TNF-α)
production by mononuclear cells, HMGB1 production by monocytes and HNP1-3
secretion by neutrophils. Naunyn-Schmiedeberg’s Archives of Pharmacology.

[145] Rejdak R, Junemann A, Grieb P (2011). Kynurenic acid and kynurenine aminotransferases in retinal aging
and neurodegeneration. Pharmacological Reports.

[146] Turski MP, Turska M, Paluszkiewicz P, Parada-Turska J, Oxenkrug GF (2013). Kynurenic Acid in the digestive system—new facts, new
challenges. International Journal of Tryptophan Research.

[147] Forrest CM, Gould SR, Darlington LG, Stone TW (2003). Levels of purine, kynurenine and lipid peroxidation products in
patients with inflammatory bowel disease. Advances in Experimental Medicine and Biology.

[148] Clarke G, Fitzgerald P, Cryan JF, Cassidy EM, Quigley EM, Dinan TG (2009). Tryptophan degradation in irritable bowel syndrome: evidence of
indoleamine 2,3-dioxygenase activation in a male cohort. BMC Gastroenterology.

[149] Christmas DM, Badawy AA-B, Hince D (2010). Increased serum free tryptophan in patients with
diarrhea-predominant irritable bowel syndrome. Nutrition Research.

[150] Julliard W, Fechner JH, Mezrich JD (2014). The aryl hydrocarbon receptor meets immunology: Friend or foe? A
little of both. Frontiers in Immunology.

[151] Stevens EA, Mezrich JD, Bradfield CA (2009). The aryl hydrocarbon receptor: A perspective on potential roles
in the immune system. The Journal of Immunology.

[152] DiNatale BC, Murray IA, Schroeder JC (2010). Kynurenic acid is a potent endogenous aryl hydrocarbon receptor
ligand that synergistically induces interleukin-6 in the presence of
inflammatory signaling. Toxicological Sciences.

[153] García-Lara L, Pérez-Severiano F, González-Esquivel D, Elizondo G, Segovia J (2015). Absence of aryl hydrocarbon receptors increases endogenous
kynurenic acid levels and protects mouse brain against excitotoxic insult
and oxidative stress. Journal of Neuroscience Research.

[154] Lanis JM, Alexeev EE, Curtis VF (2017). Tryptophan metabolite activation of the aryl hydrocarbon receptor
regulates IL-10 receptor expression on intestinal epithelia. Mucosal Immunology.

[155] van Baren N, Van den Eynde BJ (2015). Tryptophan-degrading enzymes in tumoral immune
resistance. Frontiers in Immunology.

[156] Dolušić E, Frédérick R (2013). Indoleamine 2,3-dioxygenase inhibitors: a patent review (2008
– 2012). Expert Opinion on Therapeutic Patents.

[157] Moon YW, Hajjar J, Hwu P, Naing A (2015). Targeting the indoleamine 2,3-dioxygenase pathway in
cancer. Journal for Immunotherapy of Cancer.

[158] Weng T, Qiu X, Wang J, Li Z, Bian J (2018). Recent discovery of indoleamine-2,3-dioxygenase 1 inhibitors
targeting cancer immunotherapy. European Journal of Medicinal Chemistry.

[159] Pantouris G, Mowat CG (2014). Antitumour agents as inhibitors of tryptophan
2,3-dioxygenase. Biochemical and Biophysical Research Communications.

[160] Platten M, von Knebel Doeberitz N, Oezen I, Wick W, Ochs K (2015). Cancer immunotherapy by targeting IDO1/TDO and their downstream
effectors. Frontiers in Immunology.

[161] Pilotte L, Larrieu P, Stroobant V (2012). Reversal of tumoral immune resistance by inhibition of tryptophan
2,3-dioxygenase. Proceedings of the National Acadamy of Sciences of the United States of
America.

[162] Jacobs KR, Castellano-González G, Guillemin GJ, Lovejoy DB (2017). Major developments in the design of inhibitors along the
kynurenine pathway. Current Medicinal Chemistry.

[163] Röhrig UF, Majjigapu SR, Vogel P, Zoete V, Michielin O (2015). Challenges in the Discovery of Indoleamine 2,3-Dioxygenase 1
(IDO1) Inhibitors. Journal of Medicinal Chemistry.

[164] Bailey CB, Wagner C (1974). Kynurenine formamidase. Purification and characterization of the
adult chicken liver enzyme and immunochemical analyses of the enzyme of
developing chicks. The Journal of Biological Chemistry.

[165] Han Q, Robinson H, Li J (2012). Biochemical identification and crystal structure of kynurenine
formamidase from Drosophila melanogaster. Biochemical Journal.

[166] Linderholm KR, Skogh E, Olsson SK (2012). Increased levels of kynurenine and kynurenic acid in the CSF of
patients with schizophrenia. Schizophrenia Bulletin.

[167] Erhardt S, Schwieler L, Imbeault S, Engberg G (2017). The kynurenine pathway in schizophrenia and bipolar
disorder. Neuropharmacology.

[168] Wu H-Q, Okuyama M, Kajii Y, Pocivavsek A, Bruno JP, Schwarcz R (2014). Targeting kynurenine aminotransferase II in psychiatric diseases:
Promising effects of an orally active enzyme inhibitor. Schizophrenia Bulletin.

[169] Koshy Cherian A, Gritton H, Johnson DE, Young D, Kozak R, Sarter M (2014). A systemically-available kynurenine aminotransferase II (KAT II)
inhibitor restores nicotine-evoked glutamatergic activity in the cortex of
rats. Neuropharmacology.

[170] Jayawickrama GS, Sadig RR, Sun G (2015). Kynurenine aminotransferases and the prospects of inhibitors for
the treatment of schizophrenia. Current Medicinal Chemistry.

[171] Stone TW, Darlington LG (2013). The kynurenine pathway as a therapeutic target in cognitive and
neurodegenerative disorders. British Journal of Pharmacology.

[172] Nematollahi A, Sun G, Jayawickrama G, Church W (2016). Kynurenine Aminotransferase Isozyme Inhibitors: A
Review. International Journal of Molecular Sciences.

[173] Jayawickrama GS, Nematollahi A, Sun G, Church WB, Guillemin GJ (2018). Improvement of kynurenine aminotransferase-II inhibitors guided
by mimicking sulfate esters. PLoS ONE.

[174] Linderholm KR, Alm MT, Larsson MK (2016). Inhibition of kynurenine aminotransferase II reduces activity of
midbrain dopamine neurons. Neuropharmacology.

[175] Shibata K, Marugami M, Kondo T (1996). In vivo inhibition of kynurenine aminotransferase activity by
isonicotinic acid hydrazide in rats. Bioscience, Biotechnology, and Biochemistry.

[176] Chouinard G, Annable L, Serrano M, Charette R (1977). A controlled study of a dopa decarboxylase inhibitor
(benserazide) in the treatment of schizophrenic patients. International Pharmacopsychiatry.

[177] Chouinard G, Annable L, Young SN, Sourkes TL (1978). A controlled study of tryptophan-benserazide in
schizophrenia. Communications in Psychopharmacology.

[178] Vilter RW (1964). The Vitamin B_6_-Hydrazide Relationship. Vitamins & Hormones.

[179] Jayawickrama GS, Nematollahi A, Sun G, Gorrell MD, Church WB (2017). Inhibition of human kynurenine amino-transferase isozymes by
estrogen and its derivatives. Scientific Reports.

[180] Garcia GE, Wirtz RA, Barr JR, Woolfitt A, Rosenbergt R (1998). Xanthurenic acid induces gametogenesis in Plasmodium, the malaria
parasite. The Journal of Biological Chemistry.

[181] Carpenedo R, Chiarugi A, Russi P (1994). Inhibitors of kynurenine hydroxylase and kynureninase increase
cerebral formation of kynurenate and have sedative and anticonvulsant
activities. Neuroscience.

[182] Chiarugi A, Carpenedo R, Molina MT, Mattoli L, Pellicciari R, Moroni F (1995). Comparison of the Neurochemical and Behavioral Effects Resulting
from the Inhibition of Kynurenine Hydroxylase and/or
Kynureninase. Journal of Neurochemistry.

[183] Chiarugi A, Carpenedo R, Moroni F (1996). Kynurenine disposition in blood and brain of mice: Effects of
selective inhibitors of kynurenine hydroxylase and of
kynureninase. Journal of Neurochemistry.

[184] Clark CJ, Mackay GM, Smythe GA, Bustamante S, Stone TW, Phillips RS (2005). Prolonged survival of a murine model of cerebral malaria by
kynurenine pathway inhibition. Infection and Immunity.

[185] Giorgini F, Huang S-Y, Sathyasaikumar KV (2013). Targeted deletion of kynurenine 3-Monooxygenase in mice a new
tool for studying kynurenine pathway metabolism in periphery and
brain. The Journal of Biological Chemistry.

[186] Tufvesson-Alm M, Schwieler L, Schwarcz R, Goiny M, Erhardt S, Engberg G (2018). Importance of kynurenine 3-monooxygenase for spontaneous firing
and pharmacological responses of midbrain dopamine neurons: Relevance for
schizophrenia. Neuropharmacology.

[187] Rojewska E, Piotrowska A, Makuch W, Przewlocka B, Mika J (2016). Pharmacological kynurenine 3-monooxygenase enzyme inhibition
significantly reduces neuropathic pain in a rat model. Neuropharmacology.

[188] Zwilling D, Huang S-Y, Sathyasaikumar KV (2011). Kynurenine 3-monooxygenase inhibition in blood ameliorates
neurodegeneration. Cell.

[189] Mole DJ, Webster SP, Uings I (2016). Kynurenine-3-monooxygenase inhibition prevents multiple organ
failure in rodent models of acute pancreatitis. Nature Medicine.

[190] Smith JR, Jamie JF, Guillemin GJ (2016). Kynurenine-3-monooxygenase: a review of structure, mechanism, and
inhibitors. Drug Discovery Therapy.

[191] Vescia A, Di Prisco G (1962). Studies on purified 3-hydroxyanthranilic acid
oxidase. The Journal of Biological Chemistry.

[192] Jin H, Zhang Y, You H (2015). Prognostic significance of kynurenine 3-monooxygenase and effects
on proliferation, migration and invasion of human hepatocellular
carcinoma. Scientific Reports.

[193] Liu C, Huang T, Lee C, Wang W, Lee H, Tseng L (2017). Kynurenine-3-monooxygenase (KMO) protein promotes triple negative
breast cancer progression. Annals of Oncology.

[194] Moroni F, Russi P, Gallo-Mezo MA, Moneti G, Pellicciari R (1991). Modulation of Quinolinic and Kynurenic Acid Content in the Rat
Brain: Effects of Endotoxins and Nicotinylalanine. Journal of Neurochemistry.

[195] Schouten M (2009). Strategy and Performance of Water Supply and Sanitation
Providers.

[196] (1995). Inhibitors of kynureninase. WO1995011878A1.

[197] Van Greevenbroek MMJ, Vermeulen VMM-J, De Bruin TWA (2004). Identification of novel molecular candidates for fatty liver in
the hyperlipidemic mouse model, HcB19. Journal of Lipid Research.

[198] Wu W, Nicolazzo JA, Wen L (2013). Expression of tryptophan 2,3-dioxygenase and production of
kynurenine pathway metabolites in triple transgenic mice and human
Alzheimer’s disease brain. PLoS ONE.

[199] Pellicciari R, Liscio P, Giacchè N (2018). α-Amino-β-carboxymuconate-Є-semialdehyde
Decarboxylase (ACMSD) Inhibitors as Novel Modulators of de Novo Nicotinamide
Adenine Dinucleotide (NAD^+^) Biosynthesis. Journal of Medicinal Chemistry.

[200] Fukuwatari T, Ohsaki S, Fukuoka S-I, Sasaki R, Shibata K (2004). Phthalate esters enhance quinolinate production by inhibiting
α-amino-β-carboxymuconate E-semialdehyde decarboxylase (ACMSD,
a key enzyme of the tryptophan pathway. Toxicological Sciences.

[201] Thirtamara-Rajamani K, Li P, Escobar Galvis ML, Labrie V, Brundin P, Brundin L (2017). Is the Enzyme ACMSD a Novel Therapeutic Target in
Parkinson’s Disease?. Journal of Parkinson’s Disease.

[202] Rajamani KT, Krzyzanowski S, Escobar Galvis ML, Brundin P, Brundin L (2017). 840. Developing a Rodent Model of Depression and
Neurodegenerative Disease by Targeting ACMSD, a Key Enzyme in the Kynurenine
Pathway. Biological Psychiatry.

[203] Srivastava S (2016). Emerging therapeutic roles for NAD^+^ metabolism in
mitochondrial and age-related disorders. linical and Translational Medicine.

[204] Van Gool F, Gallí M, Gueydan C (2009). Intracellular NAD levels regulate tumor necrosis factor protein
synthesis in a sirtuin-dependent manner. Nature Medicine.

[205] Frederick D, Loro E, Liu L (2016). Loss of NAD homeostasis leads to progressive and reversible
degeneration of skeletal muscle. Cell Metabolism.

[206] Ishidoh K, Kamemura N, Imagawa T, Oda M, Sakurai J, Katunuma N (2010). Quinolinate phosphoribosyl transferase, a key enzyme in de novo
NAD^+^ synthesis, suppresses spontaneous cell death by
inhibiting overproduction of active-caspase-3. Biochimica et Biophysica Acta (BBA) - Molecular Cell Research.

[207] Chuenchor W, Chang K, Doukov TI, Gerratana B (2011). Structural and kinetic characterization of NAD. Faseb J.

[208] Zhai RG, Rizzi M, Garavaglia S (2009). Nicotinamide/nicotinic acid mononucleotide adenylyltransferase,
new insights into an ancient enzyme. Cellular and Molecular Life Sciences.

[209] Verghese PB, Sasaki Y, Yang D (2011). Nicotinamide mononucleotide adenylyl transferase 1 protects
against acute neurodegeneration in developing CNS by inhibiting
excitotoxic-necrotic cell death. Proceedings of the National Acadamy of Sciences of the United States of
America.

[210] Tan B, Young DA, Lu Z (2013). Pharmacological inhibition of nicotinamide
phosphoribosyltransferase (NAMPT), an enzyme essential for NAD^+^
biosynthesis, in human cancer cells: metabolic basis and potential clinical
implications. The Journal of Biological Chemistry.

[211] Zhang C, Tong J, Huang G (2013). Nicotinamide phosphoribosyl transferase (Nampt) is a target of
microRNA-26b in colorectal cancer cells. PLoS ONE.

[212] Kraus D, Yang Q, Kong D (2014). Nicotinamide *N*-methyltransferase knockdown
protects against diet-induced obesity. Nature.

[213] Bricker AL, Carey VJ, Wessels MR (2005). Role of NADase in virulence in experimental invasive group A
streptococcal infection. Infection and Immunity.

[214] Tatsuno I, Isaka M, Minami M, Hasegawa T (2010). NADase as a target molecule of in vivo suppression of the
toxicity in the invasive M-1 group A Streptococcal isolates. BMC Microbiology.

[215] Wall KA, Klis M, Kornet J (1998). Inhibition of the intrinsic NAD^+^ glycohydrolase
activity of CD38 by carbocyclic NAD analogues. Biochemical Journal.

[216] Morales JC, Li L, Fattah FJ (2014). Review of poly (ADP-ribose) polymerase (PARP) mechanisms of
action and rationale for targeting in cancer and other
diseases. Critical Reviews in Eukaryotic Gene Expression.

[217] Curtin NJ, Szabo C (2013). Therapeutic applications of PARP inhibitors: anticancer therapy
and beyond. Molecular Aspects of Medicine.

